# Psychometric evaluation of the HFDD, PROMIS SD SF 8b, and MENQOL questionnaire in women experiencing vasomotor symptoms associated with menopause

**DOI:** 10.1186/s41687-025-00875-4

**Published:** 2025-05-07

**Authors:** Andrew Trigg, Melissa Barclay, Sophie Whyman, Asha Lehane, Helena Bradley, Christoph Gerlinger, Christian Seitz, Adam Gater, Claudia Haberland

**Affiliations:** 1 Medical Affairs Statistics, Bayer plc, Reading, UK; 2 Adelphi Values, Cheshire, UK; 3 Bayer AG, Berlin, Germany; 4 Department of Gynecology, Obstetrics and Reproductive Medicine, University Medical School of Saarland, Homburg/Saar, Germany; 5 Institute of Clinical Pharmacology and Toxicology, Charité– Universitätsmedizin Berlin, Berlin, Germany

**Keywords:** Psychometric, Vasomotor symptoms, Menopause, Sleep disturbance, Diary, PROMIS, MENQOL

## Abstract

**Background:**

Vasomotor symptoms (VMS; hot flashes) associated with menopause have significant impacts on health-related quality of life and are a leading cause for women seeking medical attention. Patient-reported outcome (PRO) instruments are commonly used to assess treatment benefit in VMS clinical trials and must demonstrate supportive evidence of measurement properties within the context of use. This study evaluated the measurement properties of scores from the Hot Flash Daily Diary (HFDD), PROMIS Sleep Disturbance Short Form 8b (PROMIS SD SF 8b) and Menopause-Specific Quality of Life (MENQOL) for measuring treatment efficacy in VMS clinical trials.

**Methods:**

Measurement properties of the HFDD, PROMIS SD SF 8b, and MENQOL scores were assessed using data (*n* = 400 participants) from a randomized, placebo-controlled, phase 3 study evaluating the efficacy and safety of elinzanetant for the treatment of VMS in postmenopausal women (OASIS 2). Analyses assessed distributional properties, reliability, validity, responsiveness, and thresholds for meaningful change.

**Results:**

Minimal floor and ceiling effects were found across the instruments at baseline. Inter-item correlations, and confirmatory factor analysis or item-response theory supported dimensionality and scoring for the MENQOL and PROMIS SD SF 8b, respectively. Test-retest reliability between Weeks 8 and 12 was good to excellent for HFDD Frequency and Severity of moderate-to-severe hot flashes scores, PROMIS SD SF 8b T-score and MENQOL Total score (intra-class correlation coefficients 0.835–0.971). Convergent and divergent correlations with instruments assessing similar or distinct constructs were consistent with pre-specified hypotheses. Known-groups validity was supported by significant differences (*p* < 0.0001) between subgroups hypothesized a priori as being clinically distinct. Responsiveness was indicated by consistent and statistically significant differences (*p* < 0.0001) in mean changes from baseline to Week 4 and 12 between groups of participants classified as ‘improved’, ‘stable’ and ‘worsened’ (effect sizes for improvement 0.81–4.62). Triangulation of estimates from multiple anchor-based analyses derived meaningful within-individual change thresholds for the HFDD, PROMIS SD SF 8b and MENQOL scores that were likely to exceed measurement error.

**Conclusions:**

Findings provide evidence that HFDD, PROMIS SD SF 8b, and MENQOL scores are valid, reliable and responsive to change, supporting their use for assessing key efficacy endpoints in VMS clinical trials.

**Supplementary Information:**

The online version contains supplementary material available at 10.1186/s41687-025-00875-4.

## Background

Vasomotor symptoms (VMS; hot flashes), sleep disturbances, and mood changes are three of the most commonly reported and bothersome symptoms associated with menopause [1] and pose substantial impacts to women’s health-related quality of life (HRQoL) [2–6]. VMS are reported by up to 80% of women during the menopausal transition and last for a median duration of 7.4 years [7]. More than 60% of postmenopausal women also report difficulties sleeping, and over 50% report feeling anxious, nervous and/or depressed [1]. Hormone therapy remains an effective option for the treatment of VMS [8]; however, many women choose not to take hormonal therapies due to concerns about side effects, medical contraindications, or for personal reasons [9–11].

Elinzanetant is a non-hormonal, selective neurokinin 1 and 3 (NK-1, 3) receptor antagonist in development for the treatment of moderate-to-severe VMS. In a phase 2 clinical study (SWITCH-1; NCT03596762) enrolling postmenopausal women experiencing moderate-to-severe VMS, elinzanetant was well-tolerated and showed significant improvements in VMS, sleep disturbance, and menopausal aspects of HRQoL [12]. Based on these results, two phase 3 studies (OASIS 1 and 2) were conducted to investigate the efficacy and safety of elinzanetant [13]. Results showed statistically significant reductions in the frequency and severity of VMS and significant improvements in sleep disturbance and menopause-related quality of life for women treated with elinzanetant compared with placebo [14].

In the OASIS 1 and 2 clinical studies, frequency and severity of VMS, sleep disturbance, and menopause-related quality of life were assessed using several patient-reported outcome (PRO) instruments, including the Hot Flash Daily Diary (HFDD), the Patient-Reported Outcomes Measurement Information System Sleep Disturbance Short Form 8b (PROMIS SD SF 8b), and the Menopause-Specific Quality of Life (MENQOL) Questionnaire [13]. For PRO instruments to be considered fit-for-purpose for supporting clinical study endpoints, regulatory guidance emphasizes the need for adequate and documented evidence of content validity, measurement properties (e.g., reliability, validity) and score interpretation in the specific context of use [15–19]. Content validity for the HFDD, PROMIS SD SF 8b, and MENQOL has been demonstrated in postmenopausal women experiencing moderate-to-severe VMS [20–22]. There is also some existing evidence supporting the measurement properties of the PROMIS SD SF 8b [23] and MENQOL [24, 25], however, no evidence is published to date supporting the measurement properties of the HFDD in the OASIS 1 and 2 target population.

The objective of this research was to evaluate the measurement properties and estimate meaningful within-individual change thresholds of scores from the HFDD, PROMIS SD SF 8b, and MENQOL to confirm their suitability to assess key efficacy endpoints in clinical studies among women experiencing moderate-to-severe VMS.

## Methods

### Study design and participants

OASIS 2 (NCT05099159) was a randomized, placebo-controlled, parallel-group, multicenter phase 3 study evaluating the efficacy and safety of elinzanetant for the treatment of moderate-to-severe VMS. The study was conducted in compliance with the International Committee on Harmonization and applicable Good Clinical Practice standards and in accordance with the Declaration of Helsinki and its later amendments. An institutional review board at each study site approved the study protocol. Women aged 40–65 years (inclusive) with a menopausal status confirmed by medical history and/or hormone levels and who experienced at least 50 moderate-to-severe hot flashes (HFs; including HFs occurring at day and night) for 7 days during screening were randomized to receive either elinzanetant 120 mg for 26 weeks or placebo for 12 weeks followed by elinzanetant 120 mg for 14 weeks (ratio of 1:1). Full details of the OASIS 2 study design and eligibility criteria have been summarized previously [13]. Data from OASIS 2 were used for the psychometric evaluation analyses described herein.

### Overview of PRO instruments subject to validation

All PROs were self-administered on a sponsor-provisioned electronic handheld device either at home or during the site visit at various timepoints. HFDD data collected at baseline and throughout the 12-week double blind treatment period, PROMIS SD SF 8b and MENQOL data collected at baseline and Weeks 4, 8, and 12, was subject to psychometric analysis. The abbreviated schedule of assessments and the list of country/language versions used in OASIS 2 are provided in Supplementary Appendix 1.

#### HFDD

The HFDD is a diary completed twice daily (morning and evening) to assess the number of mild (sensation of heat without sweating), moderate (sensation of heat with sweating, but able to continue activity), and severe (sensation of heat with sweating, causing cessation of activity) HFs experienced during the night and during the day. It also contains items assessing the number of night-time awakenings (NTAs); and the severity of sleep disturbances due to HFs at night. For assessing HF frequency and severity, the HFDD utilizes the following response types: (1) dichotomous ‘yes’ or ‘no’ for items assessing whether HFs are experienced during the day or night; (2) scroller fields to capture responses to items assessing the number of HFs experienced of differing severities.

To calculate the HFDD frequency of moderate-to-severe HF score during the treatment period, data was aggregated across seven days to a mean daily frequency (total number of moderate to severe HF during that week) / (total number of available days with data during that week). Higher scores indicate greater frequency of moderate-to-severe HFs (0 indicates no HFs); there is no upper bound for number of HFs a participant can record. To calculate the HFDD severity of moderate-to-severe HF score during the treatment period, data was aggregated across seven days by averaging the mean daily severity of HF of the available days that week: [(1 x number of mild HF) + (2 x number of moderate HF) + (3 x number of severe HF)] / (total number of mild, moderate and severe HFs on that day). Higher scores indicate greater HF severity (range 0 to 3). The HFDD scoring follows requests to assess the frequency and severity of moderate-to-severe HFs reported during a 24-hour period and aligns with regulatory recommendations for as assessing key efficacy endpoints [26].

The HFDD frequency of moderate-to-severe HF (herein referred to as the HFDD Frequency score) and HFDD severity of moderate-to-severe HF (herein referred to as the HFDD Severity score) were used to assess primary and key secondary endpoints of OASIS 2 and were subject to psychometric evaluation. The HFDD Frequency of NTAs and HFDD Sleep disturbance scores were used to assess exploratory endpoints and were utilized to evaluate convergent/divergent hypotheses for psychometric analyses.

#### PROMIS SD SF 8b

The PROMIS SD SF 8b v1.0 is an 8-item instrument designed to assess self-reported perceptions of sleep disturbances over the past seven days in adults [27]. The instrument evaluates: difficulties and concerns with falling asleep, staying asleep, and getting enough sleep; and perceptions on the quality and satisfaction of sleep. A 0–5 verbal rating scale (VRS) is used for all items (‘not at all’, ‘never’ or ‘very poor’ to ‘very much’, ‘always’ or ‘very good’). Four items are reverse-scored. The individual items for each participant are summed to form a raw total score (ranging from 8 to 40) which are then converted to T-scores (range 28.9 to 76.5) e.g., using a conversion table from the Sleep Disturbance Scoring Manual. Higher scores indicate greater sleep disturbance. The PROMIS SD SF 8b T-score was used to assess a key secondary endpoint in OASIS 2 and was subject to psychometric evaluation.

#### MENQOL

The MENQOL was developed to assess HRQoL in menopausal women [28]. It comprises 29 items across four domains of symptoms and functioning: VMS, psychosocial functioning, physical functioning, and sexual functioning. For each item, the participant indicates if they have experienced the problem in the past week (yes/no). If the participant selects ‘yes’, they rate how bothered they were by the problem using a 7-point numeric rating scale, with response options ranging from 0=‘not at all bothered’ to 6=‘extremely bothered’. If the participant selects ‘no’, no further rating is required for the given item. Individual item raw scores are converted to scores ranging from 1 to 8, where higher scores indicate greater bother. Domain scores are calculated by averaging converted individual scores. Total score is calculated as the mean of the domain scores. Data collected for the MENQOL Total score was used to assess a key secondary endpoint in OASIS 2 and was subject to psychometric evaluation. The MENQOL VMS and Sexual domain scores were used to assess exploratory endpoints and were utilized to evaluate convergent/divergent hypotheses for psychometric analyses.

### Other instruments

Five PRO instruments were used to assess exploratory endpoints and to support evaluation of the measurement properties of the HFDD Frequency score, HFDD Severity score, PROMIS SD SF 8b T-score, and MENQOL Total score. All were completed electronically by participants at home or during selected in-person visits at baseline (except for the patient global impression of change [PGI-C]) and Weeks 4, 8 and 12.

#### Patient global impression of severity (PGI-S)

Three single-item PGI-S instruments were developed to correspond with the concepts of HF frequency, HF severity, and sleep disturbances, in line with FDA recommendations [17, 18, 29]. Each PGI-S asks participants to choose the response that best describes how often, or severe their hot flashes/sleep disturbances have been over the past week. A 5-point VRS is used for the HF frequency PGI-S item (‘no hot flashes’, ‘rarely’, ‘sometimes’, ‘often’, ‘very often’) and for the HF severity and sleep disturbance PGI-S items (‘no hot flashes/sleep disturbances’, ‘mild’, ‘moderate’, ‘severe’, ‘very severe’).

#### Patient global impression of change (PGI-C)

Three single-item PGI-C instruments were developed to correspond with the concepts of HF frequency, HF severity and sleep disturbances, in line with FDA recommendations [17, 18, 29]. Each PGI-C asks participants to choose the response that best describes the overall change in the frequency or severity of hot flashes/sleep disturbances since taking the study medication. A 5-point VRS is used for the HF frequency PGI-C item (‘much less’, ‘a little less’, ‘the same [no change]’, ‘a little more’, ‘much more’ and for the HF severity and sleep disturbance PGI-C items (‘much better’ ‘a little better’, ‘the same [no change]’, ‘a little worse’, ‘much worse’).

#### EQ-5D-5 L

The EQ-5D-5L [30] is a self-administered preference-based generic instrument of health status, which includes five dimensions: mobility, self-care, usual activities, pain/discomfort, and anxiety/depression. For the EQ-5D-5L descriptive system, participants provide a rating for each question on a 5-point Likert scale based on their health ‘today’: ‘having no problems’, ‘having slight problems’, ‘having moderate problems’, ‘having severe problems’, and ‘being unable to do’/‘having extreme problems’. Participants are also asked to self-rate their own health ‘today’ on a vertical 0-100 unit visual analogue scale (0=‘worst health you can imagine’, 100=‘best health you can imagine’).

#### Insomnia severity index (ISI)

The ISI is a seven-item instrument that quantifies participants’ perception of insomnia severity, along with the impact of insomnia on daytime functioning, in the past two weeks [31–36]. The items refer to severity of difficulties in sleep onset, sleep maintenance, and early morning wakening, satisfaction with sleep pattern, noticeability of sleep problems by others, distress caused by sleep difficulties, and interference of sleep difficulties with daytime functioning. Items are scored on a five-point Likert scale from 0 (‘none’, ‘very satisfied’, ‘not at all noticeable’, ‘not at all worried’, or ‘not at all interfering’) to 4 (‘very severe’, ‘very dissatisfied’, ‘very much noticeable’, very much worried’, or ‘very much interfering’). Scores for each item are summed to produce a Total score (maximum 28), defined as 0–7 ‘no clinically significant insomnia’, 8–14 ‘subthreshold insomnia’, 15–21 ‘clinical insomnia (moderate severity)’, and 22–28 ‘clinical insomnia (severe)’ [31].

#### Beck depression inventory (BDI-II)

The BDI-II is an established and widely used instrument for assessment of depression [37–39] and includes assessment of emotional aspects reported to be experienced by postmenopausal women (e.g., sadness, irritability, loss of interest) [20, 22, 40, 41]. It consists of 21 items to assess the severity of depression over the past 2 weeks. Each item comprises a list of four statements organized by increasing level of severity regarding a particular symptom of depression. Items use a 4-point verbal response scale, ranging from 0=‘Not at all’ to 3=‘Extreme form of each symptom’. The total score ranges from 0 to 63, where a higher score indicates greater depression. Scores of 0–13 indicate minimal depression, scores of 14–19 indicate mild depression, scores of 20–28 indicate moderate depression, and scores of 29–63 indicate severe depression [42, 43].

### Psychometric analyses

Table 1 presents the main psychometric analyses performed. All psychometric analyses were detailed a priori in a psychometric analysis plan. The psychometric analysis plan details additional analyses not reported in this manuscript; interested readers can contact the corresponding author for further details. The full analysis population (comprised of all randomized participants) was used for psychometric analyses, which consisted of OASIS 2 data pooled across treatment arms. Missing data handling is provided in Supplementary Appendix 1. The adequacy of the sample size was evaluated using monte-carlo simulations and deemed sufficient for the psychometric evaluation (see Supplementary Appendix 1). All analyses used SAS version 9.4 (SAS Institute Inc., Cary, NC, USA) and ValidR version 3.5.2 (Mango Solutions Ltd., UK).

**Table 1:** Summary of psychometric analyses performed

Analysis	Description
**Descriptive statistics**	
Daily completion rates	• Daily completion rates were evaluated for the HFDD at baseline, Weeks 4, 8 and 12, to identify any unexpectedly high levels of missing data.
Distributional properties	• Floor and ceiling effects were examined at baseline and Weeks 4, 8 and 12. • A floor effect referred to the proportion of participants with a score indicating worst possible health and a ceiling effect referred to the proportion of participants with a score indicating best possible health. A threshold of 15% was used to define a notable floor or ceiling effect [48]. • Only ceiling effects (those with no HFs) were relevant for the HFDD Frequency score as there was no upper bound for the number of HFs a participant could record.
**Reliability**	
Test-retest reliability	• Test-retest reliability was evaluated by examining the stability of scores between Weeks 8 and 12 in participants defined as ‘stable’ based on relevant PGI-S anchors. The interval Week 8 to 12 was selected based on the a-priori assumption that the symptoms of participants would have been most stable during this time and based on the availability of PGI-S data (see Supplementary Materials 1 for the SoA). • Intra-class correlation coefficients (ICCs) were calculated and evaluated using pre-specified cut-off criteria: <0.50 indicating poor reliability, 0.50–0.75 indicating moderate reliability, 0.75–0.90 indicating good reliability, and > 0.90 indicating excellent reliability [59].
Composite reliability	• Composite reliability, concerned with the reliability across the different items within a multi-item score, for the PROMIS SD SF 8b was informed by the test information function from an item response theory (IRT) model using data at Week 8. The Week 8 visit was selected based on the a-priori assumption that variability between participants in item responses would be greatest at this time. • As the MENQOL Physical Domain is assumed to be a composite indicator model, based on agreement with assumptions made in prior research [24], composite reliability was not considered appropriate for the Physical Domain, or the MENQOL Total score (because it comprises the Physical Domain). However, Cronbach’s alpha and omega coefficients were produced in an exploratory manner at Week 8 (and not compared to any thresholds of acceptability), to enable comparison with past psychometric studies; results are provided in Supplementary Appendix 2.
**Validity**	
Inter-item correlations	• Item responses for the PROMIS SD SF 8b and MENQOL were (separately) correlated at Week 8. Polychoric coefficients were calculated for the PROMIS SD SF 8b and Spearman’s coefficients were calculated for the MENQOL. The Week 8 visit was selected based on the a-priori assumption that variability between participants in item responses would be greatest at this time. • Items that correlated very highly with one another (≥ 0.90) were flagged for review as they may indicate redundancy [60].
Confirmatory factor analysis (CFA)	• CFA was conducted using data from Week 8 to confirm the structure of the MENQOL. A second order model was specified per previous analyses [24, 25]. For items in the VMS, Psychosocial, and Sexual domains, item responses were modelled directly as continuous variables, with a latent variable representing each domain, and items in the Physical domain were averaged to form a single observed variable, due to the assumption this is a composite indicator model [24, 25]. The Week 8 visit was selected based on the a-priori assumption that variability between participants in item responses would be greatest at this time. • The model was estimated using maximum likelihood with robust standard errors. Standardised loadings > 0.40 considered salient. Several goodness-of-fit statistics evaluated the model including comparative fit index (> 0.95 acceptable), root mean square error of approximation (< 0.08 acceptable), standardized root mean square residual (< 0.1 acceptable), Satorra-Bentler scaled model chi-square, and Tucker-Lewis index (0.95 acceptable) [61, 44, 62, 45].
Item response theory (IRT)	• PROMIS SD SF 8b items were analyzed using a unidimensional graded response model with logit link function (as per previous development of the instrument) using Week 8 data [63, 64]. IRT was not conducted for the HFDD Frequency and HFDD Severity scores as each is measured by a single item, and CFA was chosen for the MENQOL instead to build upon previous analysis conducted by Bushmakin et al. [24] and Schultz et al. [25]. The Week 8 visit was selected based on the a-priori assumption that variability between participants in item responses would be greatest at this time. • The model was estimated by maximizing the marginal likelihood using a quasi-Newton algorithm. Item discrimination parameters (point estimates > 1 considered supportive) [64], item difficulty parameters and Yen’s Q3 statistics (values > 0.50 indicative of significant local dependence) [64] were extracted. The following plots were produced to visualize the data: (1) category characteristic curves; (2) item information functions; and (3) test information function (with horizontal reference lines at 3.33 [reliability of 0.70], 5 [reliability of 0.80], and 10 [reliability of 0.90]) [65, 66].
Convergent and divergent evidence	• Convergent and divergent hypotheses were evaluated at Week 8 by calculating Polyserial and Spearman’s correlations of the HFDD Frequency score, HFDD Severity score, PROMIS SD SF 8b T-score and MENQOL Total score with scores from instruments assessing similar and dissimilar concepts. The Week 8 visit was selected based on the a-priori assumption that variability between participants in scores would be greatest at this time. • To explore convergent validity, anchor measures were selected based on the similarity of concepts assessed to the target PRO scores (i.e., the HFDD and MENQOL VMS domain both assessing VMS). For divergent validity, anchor measures were selected where no theoretical relationship was to be expected with the target PRO scores (i.e., the HFDD and MENQOL Sexual domain or the PROMIS SD SF 8b and EQ-5D-5 L Self-care). • Hypotheses were based on a substantial correlation between similar concepts (convergent hypotheses; *r* > 0.50), [49] relatively lower correlations between distally related concepts (*r* > 0.40 or *r* > 0.30), and low correlations between differing concepts (divergent validity; *r* < 0.30). Convergent validity was considered adequate if > 75.0% of hypotheses were correct [48, 49].
Known-groups evidence	• The HFDD Frequency score, HFDD Severity score, PROMIS SD SF 8b T-score and MENQOL Total score were compared between subgroups of participants hypothesized to differ on the concept of interest (i.e., clinically distinct groups hypothesized *a priori)* at Week 8. The Week 8 visit was selected based on the a-priori assumption that variability between participants in item responses would be greatest at this time. • Known-groups were defined using PGI-S Frequency, PGI-S Severity, and PGI-S Sleep (considered clinically distinct based on clinical judgement), and ISI Total scores (considered clinically distinct based on published thresholds) for relevant target PRO scores [31]. • The following cut-offs were used to interpret the magnitude of each effect size (ES): small (ES = 0.20), moderate (ES = 0.50), large (ES = 0.80) [67]. • The statistical significance (*p* ≤ 0.50) of difference in scores between groups were calculated using a non-parametric Kruskal-Wallace test.
**Responsiveness**	
Ability to detect change	• Responsiveness was assessed by evaluating mean change in scores in groups categorized as ‘improved’, ‘stable’, or ‘worsened’ according to change from baseline (PGI-S) or scores (PGI-C) at Week 4 or 12. Changes from baseline to Weeks 4 and 12 were selected to align with key efficacy endpoints in the OASIS studies. • Spearman’s or Polyserial correlations between scores of convergent instruments and the target PRO change from baseline scores were evaluated and compared against pre-defined hypotheses. • Within-group effect sizes were calculated to evaluate the magnitude of changes (small; ES = 0.20, moderate; ES = 0.50, large; ES = 0.80) [67]. At least moderate ESs were hypothesized within the ‘improved’ groups. The statistical significance (*p* ≤ 0.50) of differences in change scores between groups were calculated using the F-test and a non-parametric Kruskal-Wallis test was also performed.
**Interpretation of scores**	
Anchor-based methods	• Score interpretation guidelines were estimated using anchor-based analyses for the HFDD Frequency score, HFDD Severity score, PROMIS SD SF 8b T-score and MENQOL Total score, using data at Week 4 and 12. Changes from baseline to Weeks 4 and 12 were selected to align with key efficacy endpoints in the OASIS studies. • Only anchor instruments that correlated > 0.3 with the PRO score of interest were used to obtain estimates [68]. PGI-S and PGI-C were used as anchor instruments. • Logistic regression (namely the ‘adjusted predictive modelling’ method of Terluin 2017) [69] and discriminant analysis (normal kernels with unequal bandwidth) [53] informed within-individual estimates. • A ‘minimally important’ threshold was targeted by creating binary variables from the PGI-S (Improved: <=-1 change; Not improved: >=0 change) and PGI-C (Improved: at least a little less/better, Not improved: the same or more/worse). To additionally target a larger ‘much improved’ threshold, the analysis was repeated with binary variable from the PGI-S (Improved: <=-2 change; Not improved: >=-1 change) and PGI-C (Improved: at least much less/better, Not improved: a little less/better, the same or more/worse). • To guide triangulation of estimates, a correlation-weighted average of estimates was calculated. [70] Correlations were z-transformed prior to their use in the correlation-weighted average.
Qualitative methods	• Estimates for meaningful within-individual change were explored through qualitative methods in a separate study, [20, 22] which were considered alongside the results of the current analyses.
Measurement error	• Minimal detectable change estimates at the 90% confidence level (MDC90) were calculated, using test-retest ICCs as reliability estimate.

## Results

### Sample characteristics

Overall, *n* = 400 participants were included in the full analysis set. Key demographic and clinical characteristics are provided in Table 2.

**Table 2:** Participant characteristics at baseline

Characteristic	Elinzanetant 120 mg (*N* = 200)	Placebo– Elinzanetant 120 mg (*N* = 200)	Total (*N* = 400)
**Race, ** ***n*** ** (%)**			
White	163 (81.5%)	172 (86.0%)	335 (83.8%)
Black or African American	35 (17.5%)	25 (12.5%)	60 (15.0%)
Asian	0	1 (0.5%)	1 (0.3%)
American Indian or Alaska Native	1 (0.5%)	1 (0.5%)	2 (0.5%)
Multiple	0	1 (0.5%)	1 (0.3%)
Not recorded	1 (0.5%)	0	1 (0.3%)
**Ethnicity**,** n (%)**			
Not Hispanic or Latino	186 (93.0%)	175 (87.5%)	361 (90.3%)
Hispanic or Latino	13 (6.5%)	24 (12.0%)	37 (9.3%)
Not reported	1 (0.5%)	1 (0.5%)	2 (0.5%)
**Age (years)**			
Mean (SD)	54.8 (5.0)	54.4 (4.5)	54.6 (4.8)
Median	55.0	54.0	54.0
Min, Max	40, 65	42, 64	40, 65
**Body Mass Index Group (kg/m2)**,** n (%)**			
< 18.5	4 (2.0%)	1 (0.5%)	5 (1.3%)
18.5 to < 25	55 (27.5%)	60 (30.0%)	115 (28.8%)
25 to < 30	78 (39.0%)	70 (35.0%)	148 (37.0%)
≥ 30	63 (31.5%)	69 (34.5%)	132 (33.0%)
**Level of education**,** n (%)**			
Missing	0	1 (0.5%)	1 (0.3%)
College or university education	97 (48.5%)	106 (53.0%)	203 (50.8%)
Professional certification	35 (17.5%)	43 (21.5%)	78 (19.5%)
Attending college	6 (3.0%)	3 (1.5%)	9 (2.3%)
Other	62 (31.0%)	47 (23.5%)	109 (27.3%)

### Descriptive statistics

Daily completion rates for the HFDD Frequency and HFDD Severity scores were high, with most participants completing all 14 days at baseline (93.3%) and all 7 days preceding Weeks 4, 8 and 12 (≥ 85.0%). No floor or ceiling effects were observed for the HFDD Frequency score, HFDD Severity score, PROMIS SD SF 8b T-score or MENQOL Total score at baseline, Weeks 4, 8 and 12.

### Reliability

Test-retest reliability results for participants defined as stable were ‘good to excellent’ (range: 0.835–0.971) for the HFDD Frequency score, HFDD Severity score, PROMIS SD SF 8b T-score and MENQOL Total score between Week 8 and Week 12 when using the PGI-S items to define stability (Table 3).

**Table 3:** Test-retest reliability for the target scores between week 8 and week 12

PRO	Stability definition	*n*	Week 8 mean (SD)	Week 12 mean (SD)	ICC estimate	95% confidence intervals	
						Lower	Upper
HFDD Frequency score	PGI-S Frequency	191	7.60 (8.553)	7.32 (8.680)	0.971	0.962	0.978
HFDD Severity score	PGI-S Severity	208	1.77 (0.772)	1.72 (0.821)	0.902	0.873	0.925
PROMIS SD SF 8b T-score	PGI-S Sleep	195	52.75 8.716)	52.55 (8.231)	0.894	0.862	0.919
MENQOL Total score	PGI-S Frequency	194	3.34 (1.267)	3.28 (1.263)	0.854	0.811	0.888
	PGI-S Severity	211	3.36 (1.275)	3.25 (1.256)	0.835	0.788	0.872

Abbreviations: CI, Confidence Interval; ICC, Intraclass Correlation Coefficient; HF, Hot flash; HFDD, Hot Flash Daily Diary; MENQOL, Menopause-Specific Quality of Life Questionnaire; PROMIS SD SF 8b, PROMIS Sleep Disturbance Short Form 8-item; SD, Standard deviation

**Fig. 1:**
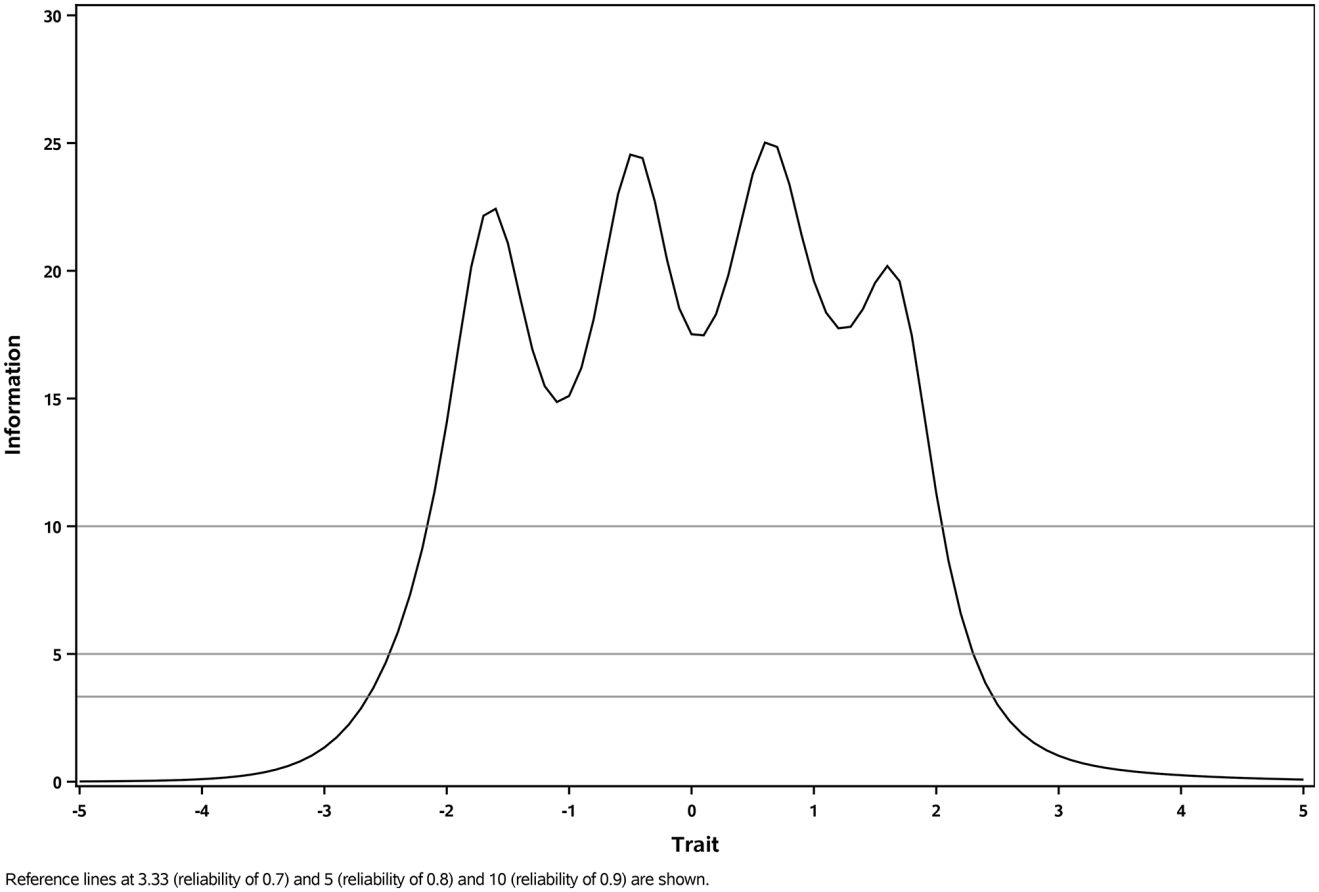
Test information curve for PROMIS SD SF 8b T-score. The test information curve shows good coverage of information across the underlying trait, indicating high reliability for most participants (reliability > 0.90 in the region of -2 to + 2)

Composite reliability of the PROMIS SD SF 8b T-score was supported by the test information function, where reliability exceeded 0.9 across the majority of the latent trait (Fig. 1).

### Validity

Inter-item correlations did not suggest any item redundancy (i.e., no correlations > 0.90) within the PROMIS SD SF 8b (Table S2) or MENQOL (Table S3).

Confirmatory factor analysis (CFA) of the pre-established MENQOL domain structure indicated that standardized factor loadings all exceeded 0.40, providing support that the MENQOL items are indicators of their respective factor (Table 4). Loadings for the second-order (Total) factor also exceeded 0.40, except for the VMS domain, (loading = 0.287). Model fit was acceptable based on root mean square error of approximation (0.053), comparative fit index (0.956) and standardized root mean square residual (0.050), but not Tucker-Lewis index (0.946) and chi-square (< 0.0001) [44, 45].

**Table 4:** Standardized factor loadings for the MENQOL at week 8

Factor	Indicator	Standardized loading	Lower 95% CI	Upper 95% CI
VMS domain	Item 1	0.823	0.749	0.897
	Item 2	0.791	0.725	0.856
	Item 3	0.872	0.822	0.921
Psychosocial domain	Item 4	0.528	0.419	0.636
	Item 5	0.700	0.624	0.776
	Item 6	0.536	0.437	0.636
	Item 7	0.724	0.661	0.786
	Item 8	0.754	0.685	0.823
	Item 9	0.681	0.602	0.760
	Item 10	0.590	0.489	0.690
Sexual domain	Item 27	0.863	0.793	0.933
	Item 28	0.587	0.487	0.686
	Item 29	0.791	0.713	0.869
Total instrument	VMS domain	0.287	0.182	0.391
	Psychosocial domain	0.711	0.568	0.853
	Physical domain	0.972	0.813	1.131
	Sexual domain	0.519	0.410	0.628

Abbreviations: CI, Confidence interval; MENQOL, Menopause-Specific Quality of Life Questionnaire; VMS, Vasomotor symptoms

**Fig. 2:**
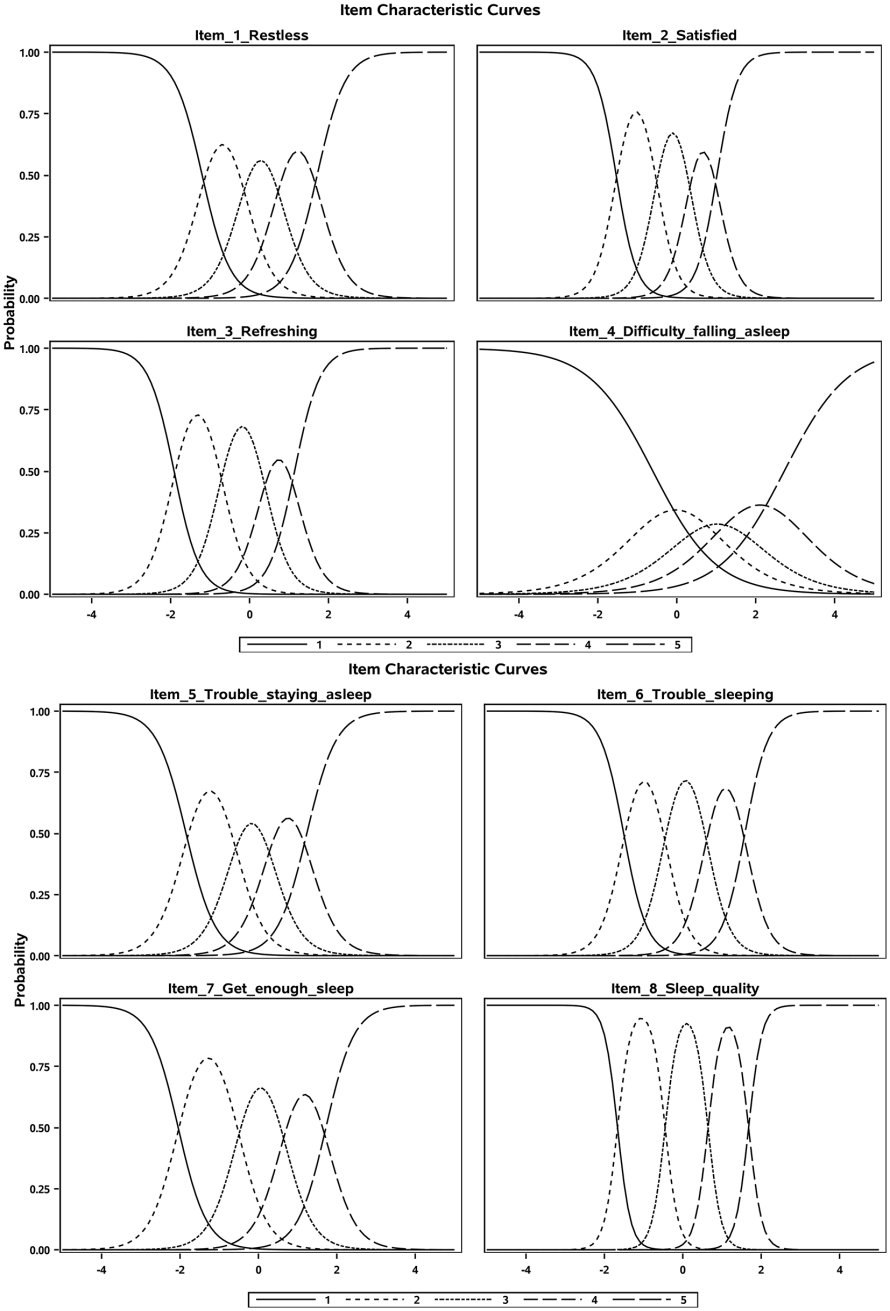
Item characteristic curves for the PROMIS SD SF 8b T-score. For all eight PROMIS SD SF 8b items, distinct peaks and relatively similar distances between thresholds are observed. The relatively less pronounced peaks for Item 4 (Difficulty falling asleep) reflect its comparatively lower discrimination parameter, however there is still a region of the latent trait where each response is the most probable, supporting its adequate performance

IRT item discrimination parameters were all > 1, supporting the ability of the PROMIS SD SF 8b items to adequately differentiate between study participants with higher and lower levels of sleep disturbance (Table S4). Yen’s Q_3_ local dependence indices were < 0.50 for all items (Table S5), providing support for the unidimensional structure of the instrument. Item characteristic curves showed distinct peaks and relatively similar distances between thresholds for all items, indicating distinct response categories and correct ordering of item responses (Fig. 2). The test information curve (Fig. 1) showed good coverage of information across the underlying trait, indicating high reliability and measurement precision of the PROMIS SD SF 8b T-score for most participants (reliability > 0.90 in the region of -2 to + 2).

A logical pattern of correlations was found when examining convergent and divergent hypotheses, with the HFDD Frequency score, HFDD Severity score, and MENQOL Total score correlating strongly with instruments assessing similar concepts and correlating weakly with instruments assessing dissimilar concepts (Table 5). The PROMIS SD SF 8b T-score also correlated as expected with all convergent or divergent instruments, except for the HFDD Sleep Disturbance score (0.499), which narrowly missed the hypothesized value for convergent evidence (> 0.5).

**Table 5:** Correlations with convergent and divergent instruments at week 8

PRO	Convergent/divergent score	Coefficient type	Hypothesized magnitude	*n*	Estimate
HFDD Frequency score	MENQOL VMS Domain	Spearman	> 0.5	349	0.688
	PGI-S Frequency	Polyserial	> 0.5	346	0.557
	MENQOL Total	Spearman	> 0.4	349	0.459
	MENQOL Sexual Domain	Spearman	< 0.3	347	0.070
HFDD Severity score	MENQOL VMS Domain	Spearman	> 0.4	349	0.655
	PGI-S Severity	Polyserial	> 0.5	346	0.741
	MENQOL Total	Spearman	> 0.3	349	0.390
	MENQOL Sexual Domain	Spearman	< 0.3	347	0.008
PROMIS SD SF 8b T-score	ISI Total	Spearman	> 0.4	359	0.842
	PGI-S Sleep	Polyserial	> 0.5	349	0.827
	EQ-5D-5 L Self-care	Polyserial	< 0.3	349	0.029
	HFDD frequency of NTAs	Spearman	> 0.5	355	0.546
	HFDD Sleep disturbance	Spearman	> 0.5	355	0.499
MENQOL Total score	HFDD Frequency	Spearman	> 0.4	349	0.459
	HFDD Severity	Spearman	> 0.3	349	0.390
	BDI-II Total	Spearman	> 0.4	350	0.636
	EQ-5D-5 L VAS	Spearman	> 0.3	349	-0.402
	PGI-S Frequency	Polyserial	> 0.4	349	0.515
	PGI-S Severity	Polyserial	> 0.3	349	0.516
	EQ-5D-5 L self-care	Polyserial	< 0.3	349	0.240

Abbreviations: BDI-II, Beck’s Depression Inventory, EQ-5D-5 L, European Quality of Life 5 Dimensions 5 Levels; HF, Hot flash; HFDD, Hot Flash Daily Diary; ISI, Insomnia Severity Index; PGI-C, Patient Global Impression of Change; PGI-S, Patient Global Impression of Severity, PROMIS SD SF 8b, PROMIS Sleep Disturbance Short Form 8-item; MENQOL, Menopause-Specific Quality of Life Questionnaire; NTAs, Night-time awakenings; VMS, Vasomotor symptoms; VAS, Visual analogue scale

Known-groups analyses indicated that there was a pattern of significantly higher (*p* < 0.0001) mean HFDD Frequency, HFDD Severity, MENQOL Total scores and the PROMIS SD SF 8b T-score for groups of participants who also scored higher (i.e., worse) on a range of anchor instruments (e.g., PGI-S and ISI) at Week 8 (Table S6). Monotonic increases were observed as expected across severity groups. Effect sizes indicated differences between adjacent groups and were moderate to large for the HFDD Frequency and Severity (ES range: 0.70–4.84) and MENQOL Total (ES range: 0.64–2.02) scores, and large for the PROMIS SD SF 8b T-score (ES range: 0.87–3.80). As such, known-groups evidence supported the ability to discriminate between groups defined by the anchor instruments.

### Responsiveness

Responsiveness correlations between scores of convergent instruments (PGI-S change from baseline scores or PGI-C scores) and target PRO change from baseline scores were consistent with prespecified hypotheses (Table S7).

Responsiveness of the HFDD Frequency score, HFDD Severity score, PROMIS SD SF 8b T-score and MENQOL Total score was supported by statistically significant differences (*p* < 0.0001 from F-test and Kruskal Wallis) in mean changes from baseline to Week 4 and 12 between groups of participants classified as ‘improved’, ‘stable’ and ‘worsened’ based on responses to PGI-S or PGI-C items, with consistently large effect sizes for improvement observed (0.81–4.62; Table 6). Negative mean change scores (indicating a reduction in the concept of interest) were also observed in the ‘stable’ and ‘worsened’ groups; however, the ‘improved’ group for each PRO score exhibited the largest reduction, as expected.

**Table 6:** Responsiveness mean changes from baseline to week 4 and week 12

Convergent instrument	Visit	Group	*n*	Mean change (SD)	Median change	Min, Max	Within-group effect size	F-test	KW test
**HFDD Frequency score**									
PGI-S Frequency	Week 4	Improved: change from Baseline <=-1	231	-9.12 (9.76)	-7.48	-75.71, 11.57	-0.89	< 0.0001	< 0.0001
		Stable: change from Baseline = 0	101	-3.34 (5.71)	-2.36	-31.31, 10.93	-0.38		
		Worsened: change from Baseline > = 1	4	-1.18 (4.02)	-1.86	-4.71, 3.71	-0.47		
	Week 12	Improved: change from Baseline <=-1	231	-9.81 (8.63)	-8.50	-75.64, 6.43	-1.04	< 0.0001	< 0.0001
		Stable: change from Baseline = 0	74	-4.40 (9.14)	-2.64	-62.71, 15.00	-0.39		
		Worsened: change from Baseline > = 1	7	-3.62 (7.74)	-1.86	-17.71, 7.21	-0.51		
PGI-C Frequency	Week 4	Improved: a little less or much less	248	-9.51 (9.70)	-7.49	-75.71, 7.64	-0.81	< 0.0001	< 0.0001
		Stable: the same (no change)	92	-2.45 (4.47)	-1.54	-20.71, 10.93	-0.33		
		Worsened: a little more or much more	23	-2.31 (8.02)	-2.00	-31.31, 11.57	-0.20		
	Week 12	Improved: a little less or much less	239	-10.14 (9.14)	-8.50	-76.14, 3.79	-0.90	< 0.0001	< 0.0001
		Stable: the same (no change)	83	-3.59 (6.24)	-2.43	-29.74, 15.00	-0.48		
		Worsened: a little more or much more	17	-9.19 (17.27)	-5.21	-62.71, 7.21	-0.52		
**HFDD Severity score**									
PGI-S Severity	Week 4	Improved: change from baseline <=-1	212	-0.84 (0.66)	-0.72	-2.77, 0.57	-3.67	< 0.0001	< 0.0001
		Stable: change from baseline = 0	105	-0.33 (0.38)	-0.26	-1.84, 0.38	-1.16		
		Worsened: change from baseline > = 1	19	-0.24 (0.33)	-0.18	-0.82, 0.24	-1.11		
	Week 12	Improved: change from baseline <=-1	227	-0.99 (0.75)	-0.87	-2.90, 0.57	-4.62	< 0.0001	< 0.0001
		Stable: change from baseline = 0	69	-0.33 (0.39)	-0.28	-1.45, 0.59	-1.04		
		Worsened: change from baseline > = 1	16	-0.26 (0.59)	-0.11	-1.94, 0.40	-1.00		
PGI-C Severity	Week 4	Improved: a little less or much less	238	-0.84 (0.66)	-0.73	-2.77, 0.68	-3.38	< 0.0001	< 0.0001
		Stable: the same (no change)	109	-0.27 (0.30)	-0.24	-1.19, 0.57	-1.11		
		Worsened: a little more or much more	16	-0.27 (0.41)	-0.22	-0.91, 0.34	-1.60		
	Week 12	Improved: a little less or much less	236	-1.03 (0.76)	-0.89	-2.90, 0.59	-4.28	< 0.0001	< 0.0001
		Stable: the same (no change)	87	-0.29 (0.37)	-0.24	-1.45, 0.30	-1.21		
		Worsened: a little more or much more	16	-0.42 (0.69)	-0.25	-1.94, 0.49	-2.15		
**PROMIS SD SF 8b T-score**									
PGI-S Sleep	Week 4	Improved: change from baseline <=-1	223	-10.18 (7.38)	-9.30	-32.60, 18.70	-1.59	< 0.0001	< 0.0001
		Stable: change from baseline = 0	97	-2.34 (5.42)	-1.20	-19.30, 9.20	-0.32		
		Worsened: change from baseline > = 1	19	-0.61 (5.26)	0.00	-12.40, 8.10	-0.09		
	Week 12	Improved: change from baseline <=-1	215	-10.50 (7.55)	-9.80	-36.70, 4.20	-1.60	< 0.0001	< 0.0001
		Stable: change from baseline = 0	81	-3.00 (4.69)	-3.10	-14.80, 7.10	-0.41		
		Worsened: change from baseline > = 1	17	-0.25 (6.35)	0.00	-13.10, 10.40	-0.03		
PGI-C Sleep	Week 4	Improved: a little less or much less	188	-11.23 (7.26)	-10.30	-32.60, 5.10	-1.80	< 0.0001	< 0.0001
		Stable: the same (no change)	146	-3.01 (5.63)	-2.40	-19.30, 18.70	-0.41		
		Worsened: a little more or much more	18	-0.84 (4.23)	-0.50	-9.80, 8.20	-0.12		
	Week 12	Improved: a little less or much less	168	-11.51 (7.71)	-11.65	-31.50, 7.20	-1.81	< 0.0001	< 0.0001
		Stable: the same (no change)	128	-4.78 (5.87)	-4.10	-36.70, 10.40	-0.67		
		Worsened: a little more or much more	30	-1.62 (5.40)	-1.05	-14.90, 8.60	-0.22		
**MENQOL Total score**									
PGI-S Frequency	Week 4	Improved: change from baseline <=-1	234	-1.34 (1.25)	-1.16	-5.01, 2.23	-1.20	< 0.0001	< 0.0001
		Stable: change from baseline = 0	101	-0.32 (0.89)	-0.26	-3.41, 1.71	-0.28		
		Worsened: change from baseline > = 1	4	-0.06 (0.44)	-0.08	-0.58, 0.49	-0.03		
	Week 12	Improved: change from baseline <=-1	231	-1.43 (1.24)	-1.35	-5.76, 2.03	-1.25	< 0.0001	< 0.0001
		Stable: change from baseline = 0	75	-0.39 (0.86)	-0.33	-2.45, 2.27	-0.35		
		Worsened: change from baseline > = 1	7	0.08 (0.83)	-0.09	-1.01, 1.10	0.06		
PGI-C Frequency	Week 4	Improved: a little less or much less	235	-1.35 (1.27)	-1.18	-5.01, 2.23	-1.13	< 0.0001	< 0.0001
		Stable: the same (no change)	92	-0.34 (0.75)	-0.26	-2.98, 1.25	-0.34		
		Worsened: a little more or much more	21	-0.31 (0.94)	-0.27	-2.47, 1.71	-0.32		
	Week 12	Improved: a little less or much less	223	-1.41 (1.30)	-1.38	-5.76, 2.27	-1.22	< 0.0001	< 0.0001
		Stable: the same (no change)	81	-0.56 (0.78)	-0.46	-2.44, 1.10	-0.52		
		Worsened: a little more or much more	17	-0.42 (1.00)	-0.25	-2.45, 1.09	-0.37		
PGI-S Severity	Week 4	Improved: change from baseline <=-1	214	-1.41 (1.22)	-1.16	-5.01, 1.17	-1.24	< 0.0001	< 0.0001
		Stable: change from baseline = 0	106	-0.38 (0.95)	-0.22	-2.83, 2.23	-0.35		
		Worsened: change from baseline > = 1	19	-0.24 (0.94)	-0.18	-1.90, 1.71	-0.22		
	Week 12	Improved: change from baseline <=-1	228	-1.40 (1.27)	-1.35	-5.76, 2.03	-1.25	< 0.0001	< 0.0001
		Stable: change from baseline = 0	69	-0.53 (0.77)	-0.44	-2.45, 1.09	-0.46		
		Worsened: change from baseline > = 1	16	-0.24 (1.24)	-0.23	-2.45, 2.27	-0.19		
PGI-C Severity	Week 4	Improved: a little less or much less	226	-1.41 (1.26)	-1.22	-5.01, 2.23	-1.18	< 0.0001	< 0.0001
		Stable: the same (no change)	108	-0.29 (0.75)	-0.26	-1.98, 1.71	-0.28		
		Worsened: a little more or much more	14	-0.42 (0.71)	-0.31	-1.90, 0.85	-0.47		
	Week 12	Improved: a little less or much less	220	-1.42 (1.31)	-1.38	-5.76, 2.27	-1.22	< 0.0001	< 0.0001
		Stable: the same (no change)	85	-0.60 (0.74)	-0.57	-2.44, 1.10	-0.55		
		Worsened: a little more or much more	16	-0.28 (0.95)	-0.13	-2.45, 1.09	-0.26		

Abbreviations: KW, Kruskal Wallis; HF, Hot Flash; HFDD, Hot Flash Daily Diary; MENQOL, Menopause-Specific Quality of Life Questionnaire; PGI-C, Patient Global Impression of Change; PGI-S, Patient Global Impression of Severity; PROMIS SD SF 8b, PROMIS Sleep Disturbance Short Form 8-item; SD, Standard Deviation

### Interpretation of scores

Anchor correlations revealed that the PGI-S (frequency, severity, sleep) and PGI-C (frequency, severity, sleep) items were sufficiently correlated with the HFDD Frequency score, HFDD Severity score, PROMIS SD SF 8b T-score and MENQOL Total score (≥ 0.30) to support meaningful change analyses. Descriptive statistics of PRO scores by anchor category, and cumulative density function plots, are provided in Supplementary material. Within-individual thresholds at Week 12 are presented in Table 7, and within-individual thresholds at Week 4 are presented in Table S16.

**Table 7:** Triangulation of anchor-based within-individual thresholds at week 12

Magnitude	Method	Anchor	Estimate (95% CI)	z-transformed correlation	Weighted average (95% CI)
**HFDD Frequency score**					
Minimal	Logistic regression	PGI-S Frequency	-5.45 (-6.67; -4.30)	0.56	-5.42 (-5.61; -5.22)
		PGI-C Frequency	-5.76 (-7.08; -4.71)	0.34	
	Discriminant analysis	PGI-S Frequency	-5.25 (-6.30; -3.92)	0.56	
		PGI-C Frequency	-5.29 (-6.43; -3.89)	0.34	
Much	Logistic regression	PGI-S Frequency	-8.24 (-9.25; -7.40)	0.56	-7.24 (-8.42; -6.05)
		PGI-C Frequency	-8.26 (-9.34; -7.51)	0.34	
	Discriminant analysis	PGI-S Frequency	-6.21 (-7.66; -5.05)	0.56	
		PGI-C Frequency	-6.24 (-7.82; -5.11)	0.34	
**HFDD Severity score**					
Minimal	Logistic regression	PGI-S Severity	-0.49 (-0.57; -0.41)	0.69	-0.59 (-0.71; -0.48)
		PGI-C Severity	-0.52 (-0.59; -0.44)	0.84	
	Discriminant analysis	PGI-S Severity	-0.77 (-0.88; -0.54)	0.69	
		PGI-C Severity	-0.60 (-0.74; -0.48)	0.84	
Much	Logistic regression	PGI-S Severity	-1.00 (-1.10; -0.90)	0.69	-0.92 (-0.97; -0.88)
		PGI-C Severity	-0.90 (-0.98; -0.82)	0.84	
	Discriminant analysis	PGI-S Severity	-0.88 (-1.02; -0.75)	0.69	
		PGI-C Severity	-0.92 (-1.04; -0.81)	0.84	
**PROMIS SD SF 8b T-score**					
Minimal	Logistic regression	PGI-S Sleep	-5.20 (-5.95; -4.34)	0.75	-6.98 (-8.29; -5.66)
		PGI-C Sleep	-7.53 (-8.24; -6.85)	0.71	
	Discriminant analysis	PGI-S Sleep	-6.96 (-8.07; -5.87)	0.75	
		PGI-C Sleep	-8.34 (-9.34; -7.37)	0.71	
Much	Logistic regression	PGI-S Sleep	-10.06 (-10.91; -9.21)	0.75	-9.82 (-10.64; -8.99)
		PGI-C Sleep	-10.93 (-11.80; -10.22)	0.71	
	Discriminant analysis	PGI-S Sleep	-9.30 (-10.56; -8.14)	0.75	
		PGI-C Sleep	-9.00 (-9.98; -7.94)	0.71	
**MENQOL Total score**					
Minimal	Logistic regression	PGI-S Frequency	-0.68 (-0.81; -0.54)	0.61	-0.94 (-1.08; -0.79)
		PGI-C Frequency	-0.82 (-0.95; -0.69)	0.60	
		PGI-S Severity	-0.75 (-0.88; -0.62)	0.55	
		PGI-C Severity	-0.84 (-0.96; -0.71)	0.57	
	Discriminant analysis	PGI-S Frequency	-0.92 (-1.12; -0.74)	0.61	
		PGI-C Frequency	-1.12 (-1.51; -0.86)	0.60	
		PGI-S Severity	-1.07 (-1.51; -0.76)	0.55	
		PGI-C Severity	-1.29 (-1.63; -1.02)	0.57	
Much	Logistic regression	PGI-S Frequency	-1.20 (-1.32; -1.08)	0.61	-1.23 (-1.33; -1.13)
		PGI-C Frequency	-1.30 (-1.43; -1.18)	0.60	
		PGI-S Severity	-1.45 (-1.61; -1.32)	0.55	
		PGI-C Severity	-1.34 (-1.46; -1.21)	0.57	
	Discriminant analysis	PGI-S Frequency	-1.03 (-1.27; -0.84)	0.61	
		PGI-C Frequency	-1.05 (-1.28; -0.84)	0.60	
		PGI-S Severity	-1.32 (-1.53; -1.13)	0.55	
		PGI-C Severity	-1.20 (-1.43; -0.99)	0.57	

Abbreviations: CI, Confidence Interval; FAS, Full Analysis Set; HF, Hot flash; HFDD, Hot Flash Daily Diary; MENQOL, Menopause-specific Quality of Life Questionnaire; PGI-C, Patient Global Impression of Change; PGI-S, Patient Global Impression of Severity; PROMIS SD SF 8b, PROMIS Sleep Disturbance Short Form 8-item

Only anchor instruments correlated > = 0.3 with the PRO score of interest are used to obtain estimates here. If correlation is < 0.3, estimate is labeled NA (not applicable). Weighted average only calculated if >1 suitable estimate available

For the HFDD Frequency score, the triangulated ‘minimally important’ within-individual thresholds were − 4.96 (Week 4) and − 5.42 (Week 12). For the HFDD Severity score, the ‘minimally important’ within-individual thresholds were − 0.55 (Week 4) and − 0.59 (Week 12). Consultation of CDF plots showed the ‘minimally important’ within-individual thresholds would correctly classify the majority of ‘improved’ participants, while correctly excluding the majority of ‘stable’ participants (see supplementary material). The triangulated anchor-based estimates for within-individual change based on ‘minimally important’ (Week 4 and 12) estimates were above minimally detectable change (MDC90) values (4.409 and 0.176 for HFDD Frequency and HFDD Severity scores, respectively). Previous qualitative cognitive debriefing of the PGI items also supports that the ‘minimally important’ thresholds can be considered meaningful to this population [20].

For the PROMIS SD SF 8b T-score, the suggested ‘minimally important’ within-individual thresholds were − 6.09 (Week 4) and − 6.98 (Week 12). The weighted average anchor-based estimates for within-individual change based on ‘minimally important’ (Week 4 and 12 estimates) were above the MDC90 (MDC90 = 5.071). For the MENQOL Total score, the suggested ‘minimally important’ within-individual thresholds were − 0.82 (Week 4) and − 0.94 (Week 12). For the MENQOL Total score (Week 4 and 12) only the ‘much improved’ thresholds (-1.27 at Week 4, -1.23 at Week 12) surpassed the MDC90 (MDC90 = 1.093). Across the scores, consultation of CDF plots showed the ‘minimally important’ within-individual thresholds would correctly classify the majority of ‘improved’ participants, while correctly excluding the majority of ‘stable’ participants (see Supplementary material).

## Discussion

The aim of this study was to evaluate psychometric properties of scores from the HFDD, PROMIS SD SF 8b, and MENQOL to provide evidence of their appropriateness for assessing efficacy endpoints in VMS clinical trials. Findings provide evidence that the HFDD Frequency score, HFDD Severity score, PROMIS SD SF 8b T-score and MENQOL Total score are reliable, valid, and responsive to change in the specific context of postmenopausal women experiencing moderate-to-severe VMS. Triangulation of estimates from multiple anchor-based analyses derived a range of ‘minimally important’ within-individual change thresholds for the HFDD and PROMIS SD SF 8b scores exceeding measurement error (only ‘much improved’ thresholds exceeded measurement error for the MENQOL Total score). Findings add to the previous qualitative evidence supporting the content validity of the HFDD, PROMIS SD SF 8b and MENQOL instruments in postmenopausal women [20, 22].

The sample of participants included in this study is considered representative of the menopausal population in the US. While Asian individuals were underrepresented in OASIS 2, this is a result of the study locations chosen and aligns with previous findings that VMS are less frequently reported, and reported to be less bothersome, by Asian women [46, 47].

Daily completion rates for the HFDD were high across all instruments, and no floor and/or ceiling effects were observed for any of the target PRO scores, indicating that the range of HFDD, PROMIS SD SF 8b and MENQOL scores was not restricted.

Test-retest reliability results for the HFDD, PROMIS SD SF 8b, and MENQOL scores met the threshold for good or excellent agreement across all timepoints, regardless of how stability was defined. Notably, test-retest reliability results for the PROMIS SD SF 8b T-score and MENQOL Total score were stronger than those observed in previous psychometric studies evaluating these instruments [23, 25].

Dimensionality of the PROMIS SD SF 8b and MENQOL was generally supported by the CFA (MENQOL) and IRT (PROMIS SD SF 8b). For CFA, the MENQOL VMS domain and Total score loaded below the pre-specified threshold of 0.40, which could potentially question the appropriateness of averaging all domain scores with equal weighting to yield a total score. However, the present domain weightings were considered appropriate as factor loadings above 0.40 for the MENQOL VMS domain have previously been documented [24, 25] and CFA results observed in this study broadly supported the pre-established MENQOL domain structure and demonstrated adequate model fit for the majority of fit statistics calculated [44, 45]. Convergent and divergent correlations with instruments of similar or distinct concepts were consistent with pre-specified hypotheses, providing evidence that the HFDD Frequency score, HFDD Severity score, PROMIS SD SF 8b T-score and MENQOL Total score were truly measuring their respective concepts of interest. The only exception to this was for the correlation between the PROMIS SD SF 8b T-score and the HFDD Sleep disturbance score, which narrowly missed the hypothesized magnitude for convergent evidence. Nonetheless, as more than 75.0% of hypotheses were met [48, 49], overall the findings support convergent and divergent validity of the PROMIS SD SF 8b T-score. Results of this study are consistent with previous research, where convergent validity of the PROMIS SD SF 8b raw score was generally supported by moderate correlations with instruments of sleep disturbance and sleep related impairment [23].

Known-groups comparisons demonstrated that the HFDD Frequency score, HFDD Severity score, PROMIS SD SF 8b T-score and MENQOL Total score can discriminate between groups considered to be clinically distinct based on external instruments of related concepts, whereby significant linear trends in the expected direction and moderate or large between-group effect sizes were consistently observed. Specifically, the four PRO scores were shown to be sensitive to improvements, with large effect sizes within groups defined as ‘improved’ and statistically significant differences between the ‘improved’, ‘stable’, and ‘worsened’ groups. Notably, for stable and worsened groups, negative means were also observed indicating improvements, contrary to what would be expected from a truly stable group (i.e., a group that had zero average change in score) or a group reporting worsening (e.g., more frequent HFs). However, sample sizes for worsened groups were generally small so results should be interpreted with caution. It should also be noted that the improved group always had a larger magnitude of within-group ES, as anticipated. Furthermore, some improvement in PRO scores within all improved, stable and worsened groups is a common observation when analyzing clinical trial data where populations are generally expected to improve in health over time [50–52]. Nevertheless, responsiveness of the HFDD Frequency score, HFDD Severity score, PROMIS SD SF 8b T-score and MENQOL Total score was supported, with findings for the PROMIS SD SF 8b T-score and MENQOL Total score aligning with previous psychometric studies evaluating these or related scores in menopausal women experiencing moderate-to-severe VMS [23, 25].

The triangulated ‘minimally important’ within-individual change thresholds for the HFDD Frequency score, HFDD Severity score, and PROMIS SD SF 8b T-score, and the ‘much improved’ within-individual change thresholds for the MENQOL Total score, were deemed robust, given there was limited variability in the threshold estimates across multiple anchors and they are highly likely to exceed measurement error. When compared with previous studies, the triangulated ‘minimally important’ within-individual thresholds suggested here for the HFDD Frequency score (Week 4: -4.96; Week 12: -5.42) are higher than what has been proposed for similar hot flash daily diaries (e.g., -2.73 at Week 4 by Gerlinger et al.) [53, 54], but more closely align with the within-individual thresholds (Week 4: -5.73/-5.79; Week 12: -6.20/-6.28) used more recently to support clinical study endpoints in women with moderate-to-severe VMS [55, 56]. Similarly, from the current analysis, the triangulated ‘much improved’ within-individual change thresholds (Week 4: -7.51; Week 12: -7.24) were higher than what has been previously proposed (-5.76 at Week 12) by Gerlinger et al. for similar hot flash daily diaries [53]. For the HFDD Severity score, the ‘minimally important’ within-individual change threshold (Week 4: -0.55; Week 12: -0.59) lies between the minimal clinically important difference (Week 4: -0.350; Week 12: -0.225) and clinically important differences (Week 4: -0.525; Week 12: -0.775) proposed by Constantine et al., used to evaluate the clinically meaningful effect of a hormone therapy in VMS [57]. In addition, from the current analysis, ‘much improved’ within-individual change thresholds were also established (Week 4: -0.80; Week 12: -0.92). Differences between the current HFDD Frequency and HFDD Severity score thresholds and previous thresholds are likely due to differences in the anchors used (prior anchor-based analyses being based on PGI-C responses [55, 56] or ‘Clinical Global Impression– Improvement’ responses [53, 54, 57] only) and analytical approaches employed to support score interpretation analyses.

For the MENQOL Total score, the triangulated ‘minimally important’ within-individual change thresholds (Week 4: -0.82; Week 12: -0.94) from this analysis were consistent with recent research by Schultz et al. [25] in a VMS indication (-0.90), which used different anchors measures and methods (i.e., anchor-based approaches together with distribution-based estimates and receiver operating characteristic curves).

There are some limitations of the current study. The sample size was generally deemed sufficient for psychometric analyses; however, for a few known-groups comparisons and responsiveness analyses, sample sizes were small in specific groupings (e.g., PGI-S Frequency ‘Worsened’ group, *n* = 4). While this limits comparisons and interpretation of these specific findings, sufficient evidence using other reference instruments supported validity and ‘Improved’ and ‘Stable’ groups comprised larger sample sizes. Convergent and divergent correlations based on the ISI, BDI-II and EQ-5D-5 L employ different recall periods to the PROMIS SD SF 8b and MENQOL which may introduce some discrepancies. Analyses may also be limited by the lack of objective, clinical and physiological assessments used as convergent instruments to evaluate the psychometric properties of instruments assessing VMS and sleep. Devices that detect differences in electrical connectivity of the skin provide a means to measure VMS objectively [58], and polysomnography is considered the gold standard to objectively measure sleep related parameters. Whilst future studies could use such physiological measures, these can be difficult to implement without limiting ecological validity. Similarly, though physiological measures provide valuable insights, studies have demonstrated discordance with self-reported instruments of HFs in particular [58], likely due to them measuring slightly different concepts (e.g., respondent perceptions of how well they have slept vs. objective indicators of sleep quantity/quality). Lastly, participants had to experience at least 50 moderate-to-severe hot flashes for 7 days during screening to be eligible for OASIS 2, which could impact the generalizability of the current findings to women who only experience mild VMS.

## Conclusion

This study provides evidence that the HFDD Frequency score, HFDD Severity score, PROMIS SD SF 8b T-score, and MENQOL Total score are reliable, valid, and responsive to change, supporting their application for assessing key efficacy endpoints in VMS clinical trials. Thresholds for meaningful within-individual change were established and supported in the context of evaluating the efficacy and safety of elinzanetant for the treatment of moderate to severe VMS associated with menopause.

## Electronic supplementary material

Below is the link to the electronic supplementary material.

Supplementary Material 1

## Data Availability

Bayer commits to sharing upon request from qualified scientific and medical researchers patient-level clinical trial data, study-level clinical trial data, and protocols from clinical trials in patients for medicines and indications approved in the United States (US) and European Union (EU) as necessary for conducting legitimate research. Details of Bayer’s clinical trial transparency policy can be found at https://clinicaltrials.bayer.com/transparency-policy/.
