# Supplementary material for: Psychometric evaluation of the HFDD, PROMIS SD SF 8b, and MENQOL questionnaire in women experiencing vasomotor symptoms associated with menopause
Source: J Patient Rep Outcomes. 2025 May 7;9:50. doi: 10.1186/s41687-025-00875-4 (PMC12058613; doi:10.1186/s41687-025-00875-4)

# Supplementary File

**Appendix 1. Methods**

Abbreviated schedule of assessments

| **Visit name** | **BL** | **T1** | **T2** | **T3** | **T4** | **T5** | **T6/EoT** | **FU** |
| --- | --- | --- | --- | --- | --- | --- | --- | --- |
| **In person (IP) or phone visit** | **IP** | **IP** | **IP** | **IP** | **IP** | **phone** | **IP** | **IP** |
| **Visit Week** | **1** | **4** | **8** | **12** | **16** | **20** | **26** | **30** |
| **Visit Day** | 1 | 22-28 | 50-56 | 85-90 | 106-112 | 134-140 | 176-182 | 204-217 |
| **Allowed window in days** |  | -7 | -7 | +6 | -7 | -7 | -7 | ±7 |
| **eDiary and other ePRO instruments** | | | | | | | | |
| HFDD (twice daily) | **----------------------------------------------------------------------------------------->** | | | | | | | |
| Sleepiness Scale (7 consecutive days, reported in the evening) | during Week 1, Week 4, and Week 12 | | | | | | |  |
| PROMIS SD SF 8b | once weekly | | ⚫ | ⚫ | ⚫ |  | ⚫ | ⚫ |
| ISI | ⚫ | ⚫ | ⚫ | ⚫ | ⚫ |  | ⚫ | ⚫ |
| MENQOL | ⚫ | ⚫ | ⚫ | ⚫ | ⚫ |  | ⚫ | ⚫ |
| BDI-II |  | ⚫ | ⚫ | ⚫ | ⚫ |  | ⚫ | ⚫ |
| EQ-5D-5L | once weekly | | ⚫ | ⚫ | ⚫ |  | ⚫ | ⚫ |
| PGI-S | once weekly | | ⚫ | ⚫ | ⚫ |  | ⚫ | ⚫ |
| PGI-C |  | ⚫ | ⚫ | ⚫ | ⚫ |  | ⚫ | ⚫ |
| **Screening and randomization** | | | | | | | | |
| Inclusion/exclusion criteria | ⚫ |  |  |  |  |  |  |  |
| Participant randomization | ⚫ |  |  |  |  |  |  |  |
| BL=baseline, eCOA = Electronic clinical outcome assessment, EoT = End of treatment, IP = in person, FU = follow up, T = treatment.  Questionnaires: BDI-II=Beck Depression Inventory, EQ-5D-5L=European Quality of Life 5-dimension 5-level questionnaire, HFDD=Hot Flash Daily Diary, ISI=Insomnia Severity Index, MENQOL=Menopause Specific Quality of Life Scale, PGI-C=Patient Global Impression of Change, PGI-S=Patient Global Impression of Severity, PROMIS SD SF 8b=Patient-reported Outcomes Measurement Information System Sleep Disturbance Short Form 8b. | | | | | | | | |

List of country/language versions produced based on the original US English version (HFDD, PROMIS SD SF 8b) or original Canada English version (MENQOL)

| **Country** | **Language** |
| --- | --- |
| Canada | English, French |
| Czech Republic | Czech |
| Germany | German |
| Italy | Italian |
| Norway | Norwegian |
| Poland | Polish |
| Portugal | Portuguese |
| Slovakia | Slovak |
| Switzerland | French, German, Italian |
| US | English, Spanish |

Sample size determination

The sample size was determined to power the primary and key secondary endpoints. The study aimed to randomize a total of 370 participants. Assuming a drop-out rate of 10% in the first 3 months, this would result in approximately 332 evaluable participants (166 per arm) who completed 12 weeks of treatment.

For the psychometric analyses, parameter stability given a sample size of 332 participants was assessed through simulations. Of all the analyses described within the psychometric analysis plan, the one theoretically requiring the largest sample size was confirmatory factor analysis (CFA) of the MENQOL. Parameter estimates of a CFA model on SWITCH-1 data (NCT03596762 2020; Simon et al., 2023) were obtained. Data was then simulated based on these SWITCH-1 parameters and OASIS 2 expected sample size of 332 (500 datasets), with a CFA model fitted to each simulated dataset. The relative bias and coverage of the loading parameter estimates, plus model convergence (based on the 500 CFA models), was assessed:

- Relative Bias was calculated as (average estimate – specified parameter)/specified parameter. It should ideally be <0.05 for the loadings (Hoogland & Boomsma, 1998), but <0.10 may be acceptable (Muthén & Muthén, 2002).
- Coverage: The percentage of 95% confidence intervals covering the parameters underlying the data. This should ideally be between 0.91 and 0.98 (Muthén & Muthén, 2002).
- Convergence: How often the model converges (i.e. is able to estimate all parameters without any issues).

For a sample size of 332 (reflecting the number of study participants expected to complete 12 weeks of treatment), the relative bias was <0.05 (range 0.01-0.020) and coverage ranged from 0.940 to 0.964 for all loading parameters. Convergence was 99.8% (i.e. 499 out of 500). Given the acceptable relative bias and coverage for the loading estimates, and high convergence rate, the sample size for the full analysis set (even with 10% dropout) is expected to be appropriate for the CFA model. This assumes that the model estimated using SWITCH-1 data is an adequate representation of the true population model; however, this assumption is supported by results of another study fitting the same model structure (Bushmakin et al., 2014). As all other psychometric analyses in this SAP theoretically require lower sample sizes than the CFA model, the sample size is deemed generally appropriate

*Bushmakin AG, Abraham L, Pinkerton JV, Cappelleri JC, Mirkin S. Evaluation of the measurement model and clinically important differences for menopause-specific quality of life associated with bazedoxifene/conjugated estrogens. Menopause. 2014;21(8):815-822.*

*Hoogland JJ, Boomsma A. Robustness studies in covariance structure modeling: An overview and a meta-analysis. Sociological Methods & Research. 1998;26(3):329-367.*

*Muthén LK, Muthén BO. How to use a Monte Carlo study to decide on sample size and determine power. Structural equation modeling. 2002;9(4):599-620*

*Simon JA, Anderson RA, Ballantyne E, et al. Efficacy and safety of elinzanetant, a selective neurokinin-1, 3 receptor antagonist for vasomotor symptoms: a dose-finding clinical trial (SWITCH-1). Menopause. 2023;30(3):239-246.*

Handling of missing data

There are two types of missing data for eDiary/ePRO measures: missing data at the ‘form’ level and missing data at the ‘item’ level. Form level missing refers to a participant missing an entire eDiary/ePRO assessment for a given time point (e.g., a given day for the HFDD and a given week for the PROMIS SD SF 8b). In general, form level data may be missing due to participant’s early withdrawal from the study, the inability to evaluate an endpoint at a particular time point, or non-compliance. There were no item level missing data for the HFDD and the PROMIS SD SF 8b because the participants had to select an answer for an item in order to move on to the next item on the device. This did not apply to the MENQOL.

**HFDD:** A diary day for the calculation of the frequency and severity of HF consists of the evening entry (evening diary) and the morning entry (morning diary) of the subsequent day. A day was considered available for the calculation of the frequency and severity of HF if at least the evening or the morning entry (of the subsequent day) was not missing. The daily number of HFs was calculated as the sum of HFs documented in both the evening and morning diary. Retrospective data entry was possible for a limited time. For the morning diary these retrospective entries were allowed on the same day between 11:00AM and 11:59PM. For the evening diary the retrospective entry option was available between midnight until 10:59AM on the day after the missed entry. If only the evening or morning entry was available, then only this was used for that particular day. For the evaluation of the frequency and severity of HF, the daily HF assessments were aggregated to a mean daily frequency and severity respectively from the data of a particular week. In case data was not available for more than 2 days within a week, the value for that particular week was set to missing. Missing weekly values were not imputed for this psychometric analysis.

**PROMIS SD SF 8b:** By design of the ePRO, there were no item level missing data for the PROMIS SD SF 8b. Missing form level values were not imputed for this psychometric analysis.

**MENQOL**: For the MENQOL, participants had the option to skip single items (e.g., if they did not feel comfortable to complete them). Missing item scores were imputed (following developer-recommended approach), if the participant had responded to more than one half of the domain items (i.e., at least two items in the VMS Domain, two items in the Sexual Domain, four items in the Psychosocial Domain and nine items in the Physical Domain). The imputed value is the mean for that item generated from the other participants who responded. For missing item scores at baseline, all participants who responded to the item were considered. For post-randomization, only the responses of the participants in the same treatment group were used. When data from other participants were used for imputation, half of the participants must have responded to the item before an imputed value was calculated. If the participant answered ‘yes’ but did not indicate ‘how bothered’ she was (partially missing data), the value would have been imputed from her own answers by calculating the mean of her ‘bothered’ scores for all her ‘yes’ answers within that domain. If she answered ‘no’ to all other domain items, the imputed score would have been generated from the mean of all the participants who responded ‘yes’ to the same item as discussed above (Lewis et al., 2005). If missing item scores within a domain could not be imputed (because participant responded to half or less than half of the domain items), the domain score was set to missing and the Total score was calculated as the mean of the remaining available domains. Missing form level values were not imputed for this psychometric analysis.

*Lewis JE, Hilditch JR, Wong CJ. Further psychometric property development of the Menopause-Specific Quality of Life questionnaire and development of a modified version, MENQOL-Intervention questionnaire. Maturitas. 2005;50(3):209-221.*

**PGI items:** No imputation of missing item or form level values was performed.

**EQ-5D-5L, ISI and BDI-II:** By design of the ePRO, there were no item level missing data. No imputation of missing form level values was performed.

**Appendix 2: Results**

Composite reliability was explored for the MENQOL VMS, Psychosocial and Sexual Domain scores at Week 8. The MENQOL VMS, Psychosocial and Sexual domain scores exhibited acceptable internal consistency reliability with alpha and omega coefficients for each score being >0.70 (Table S1). Though only produced on an exploratory basis, coefficients for the MENQOL Physical Domain and Total scores were also high (>0.80).

Table S1. Composite reliability for MENQOL scores at Week 8

| Score | n | Coefficient | Estimate | Lower 95% CI | Upper 95% CI |
| --- | --- | --- | --- | --- | --- |
| MENQOL Total | 342 | alpha | 0.907 | 0.891 | 0.920 |
|  |  | omega total | 0.907 | 0.889 | 0.920 |
| MENQOL Sexual Domain | 347 | alpha | 0.786 | 0.739 | 0.827 |
|  |  | omega total | 0.794 | 0.752 | 0.833 |
| MENQOL VMS Domain | 349 | alpha | 0.863 | 0.831 | 0.892 |
|  |  | omega total | 0.865 | 0.833 | 0.894 |
| MENQOL Physical Domain | 350 | alpha | 0.878 | 0.856 | 0.894 |
|  |  | omega total | 0.875 | 0.851 | 0.893 |
| MENQOL Psychosocial Domain | 352 | alpha | 0.827 | 0.792 | 0.855 |
|  |  | omega total | 0.833 | 0.799 | 0.859 |
| Abbreviations: CI, confidence interval; FAS, Full Analysis Set; MENQOL, Menopause-Specific Quality of Life Questionnaire; VMS, vasomotor symptoms Omega total coefficient is based on a unidimensional model fitted using maximum likelihood. Alpha coefficient is calculated based on observed item covariances. Confidence intervals derived through bootstrapping (1000 samples with replacement). | | | | | |

**Table S2. Inter-item polychoric correlation coefficients for the PROMIS SD SF 8b T-score at Week 8**

| Item | 1 | 2 | 3 | 4 | 5 | 6 | 7 | 8 |
| --- | --- | --- | --- | --- | --- | --- | --- | --- |
| 1: Restless | 1.000 | 0.734 | 0.661 | 0.616 | 0.768 | 0.806 | 0.618 | 0.765 |
| 2: Satisfied | 0.734 | 1.000 | 0.822 | 0.457 | 0.709 | 0.749 | 0.765 | 0.864 |
| 3: Refreshing | 0.661 | 0.822 | 1.000 | 0.442 | 0.662 | 0.698 | 0.759 | 0.852 |
| 4: Difficulty falling asleep | 0.616 | 0.457 | 0.442 | 1.000 | 0.464 | 0.638 | 0.460 | 0.516 |
| 5: Trouble staying asleep | 0.768 | 0.709 | 0.662 | 0.464 | 1.000 | 0.816 | 0.659 | 0.770 |
| 6: Trouble sleeping | 0.806 | 0.749 | 0.698 | 0.638 | 0.816 | 1.000 | 0.685 | 0.814 |
| 7: Get enough sleep | 0.618 | 0.765 | 0.759 | 0.460 | 0.659 | 0.685 | 1.000 | 0.814 |
| 8: Sleep quality | 0.765 | 0.864 | 0.852 | 0.516 | 0.770 | 0.814 | 0.814 | 1.000 |
| Polychoric correlation coefficients, using pairwise deletion. Abbreviations: PROMIS SD SF 8b, PROMIS Sleep Disturbance Short Form 8-item  Shading: r≥0.3, r≥0.5, N/A as same item. | | | | | | | | |

**Table S3. Inter-item Spearman’s correlation coefficients for the MENQOL at Week 8**

| **Domain** | **Item** | **1** | **2** | **3** | **4** | **5** | **6** | **7** | **8** | **9** | **10** | **11** | **12** | **13** | **14** | **15** | 16 |
| --- | --- | --- | --- | --- | --- | --- | --- | --- | --- | --- | --- | --- | --- | --- | --- | --- | --- |
| **VMS** | **1** | 1.000 | 0.696 | 0.758 | 0.073 | 0.061 | 0.128 | 0.178 | 0.001 | 0.116 | 0.085 | 0.038 | 0.221 | 0.238 | 0.554 | 0.111 | 0.110 |
|  | **2** | 0.696 | 1.000 | 0.739 | 0.101 | 0.081 | 0.088 | 0.180 | 0.026 | 0.144 | 0.119 | 0.008 | 0.183 | 0.237 | 0.566 | 0.111 | 0.126 |
|  | **3** | 0.758 | 0.739 | 1.000 | 0.004 | 0.028 | 0.053 | 0.168 | -0.054 | 0.051 | 0.049 | 0.019 | 0.191 | 0.236 | 0.495 | 0.076 | 0.100 |
| **Psychosocial** | **4** | 0.073 | 0.101 | 0.004 | 1.000 | 0.370 | 0.207 | 0.390 | 0.489 | 0.237 | 0.436 | 0.156 | 0.144 | 0.264 | 0.226 | 0.159 | 0.251 |
|  | **5** | 0.061 | 0.081 | 0.028 | 0.370 | 1.000 | 0.344 | 0.417 | 0.617 | 0.506 | 0.411 | 0.303 | 0.203 | 0.373 | 0.199 | 0.153 | 0.359 |
|  | **6** | 0.128 | 0.088 | 0.053 | 0.207 | 0.344 | 1.000 | 0.440 | 0.302 | 0.428 | 0.253 | 0.366 | 0.276 | 0.370 | 0.172 | 0.261 | 0.352 |
|  | **7** | 0.178 | 0.180 | 0.168 | 0.390 | 0.417 | 0.440 | 1.000 | 0.508 | 0.494 | 0.366 | 0.327 | 0.314 | 0.541 | 0.263 | 0.271 | 0.602 |
|  | **8** | 0.001 | 0.026 | -0.054 | 0.489 | 0.617 | 0.302 | 0.508 | 1.000 | 0.449 | 0.438 | 0.257 | 0.197 | 0.387 | 0.128 | 0.196 | 0.440 |
|  | **9** | 0.116 | 0.144 | 0.051 | 0.237 | 0.506 | 0.428 | 0.494 | 0.449 | 1.000 | 0.381 | 0.273 | 0.335 | 0.473 | 0.192 | 0.310 | 0.451 |
|  | **10** | 0.085 | 0.119 | 0.049 | 0.436 | 0.411 | 0.253 | 0.366 | 0.438 | 0.381 | 1.000 | 0.239 | 0.180 | 0.366 | 0.190 | 0.227 | 0.296 |
| **Physical** | **11** | 0.038 | 0.008 | 0.019 | 0.156 | 0.303 | 0.366 | 0.327 | 0.257 | 0.273 | 0.239 | 1.000 | 0.349 | 0.351 | 0.121 | 0.307 | 0.269 |
|  | **12** | 0.221 | 0.183 | 0.191 | 0.144 | 0.203 | 0.276 | 0.314 | 0.197 | 0.335 | 0.180 | 0.349 | 1.000 | 0.433 | 0.246 | 0.507 | 0.487 |
|  | **13** | 0.238 | 0.237 | 0.236 | 0.264 | 0.373 | 0.370 | 0.541 | 0.387 | 0.473 | 0.366 | 0.351 | 0.433 | 1.000 | 0.441 | 0.371 | 0.543 |
|  | **14** | 0.554 | 0.566 | 0.495 | 0.226 | 0.199 | 0.172 | 0.263 | 0.128 | 0.192 | 0.190 | 0.121 | 0.246 | 0.441 | 1.000 | 0.169 | 0.249 |
|  | **15** | 0.111 | 0.111 | 0.076 | 0.159 | 0.153 | 0.261 | 0.271 | 0.196 | 0.310 | 0.227 | 0.307 | 0.507 | 0.371 | 0.169 | 1.000 | 0.449 |
|  | **16** | 0.110 | 0.126 | 0.100 | 0.251 | 0.359 | 0.352 | 0.602 | 0.440 | 0.451 | 0.296 | 0.269 | 0.487 | 0.543 | 0.249 | 0.449 | 1.000 |
|  | **17** | 0.145 | 0.161 | 0.164 | 0.269 | 0.340 | 0.321 | 0.637 | 0.445 | 0.508 | 0.327 | 0.314 | 0.436 | 0.586 | 0.303 | 0.376 | 0.757 |
|  | **18** | 0.193 | 0.187 | 0.158 | 0.352 | 0.357 | 0.391 | 0.624 | 0.430 | 0.494 | 0.371 | 0.360 | 0.480 | 0.726 | 0.327 | 0.373 | 0.671 |
|  | **19** | 0.114 | 0.092 | 0.108 | 0.185 | 0.232 | 0.297 | 0.260 | 0.207 | 0.269 | 0.152 | 0.306 | 0.243 | 0.290 | 0.102 | 0.327 | 0.330 |
|  | **20** | 0.120 | 0.108 | 0.110 | 0.170 | 0.182 | 0.199 | 0.309 | 0.278 | 0.244 | 0.181 | 0.279 | 0.259 | 0.308 | 0.163 | 0.248 | 0.397 |
|  | **21** | 0.076 | 0.064 | 0.077 | 0.098 | 0.144 | 0.129 | 0.203 | 0.184 | 0.186 | 0.133 | 0.190 | 0.126 | 0.166 | 0.121 | 0.176 | 0.190 |
|  | **22** | 0.124 | 0.051 | 0.098 | 0.204 | 0.150 | 0.277 | 0.266 | 0.141 | 0.196 | 0.167 | 0.249 | 0.260 | 0.253 | 0.153 | 0.264 | 0.317 |
|  | **23** | 0.138 | 0.113 | 0.158 | 0.207 | 0.252 | 0.289 | 0.346 | 0.275 | 0.253 | 0.228 | 0.527 | 0.313 | 0.382 | 0.184 | 0.273 | 0.297 |
|  | **24** | 0.116 | 0.148 | 0.115 | 0.193 | 0.162 | 0.168 | 0.301 | 0.256 | 0.230 | 0.139 | 0.179 | 0.495 | 0.358 | 0.189 | 0.466 | 0.464 |
|  | **25** | 0.128 | 0.138 | 0.119 | 0.116 | 0.183 | 0.180 | 0.241 | 0.179 | 0.174 | 0.217 | 0.222 | 0.293 | 0.274 | 0.224 | 0.226 | 0.282 |
|  | **26** | 0.043 | 0.054 | 0.065 | 0.103 | 0.074 | 0.119 | 0.194 | 0.160 | 0.151 | 0.110 | 0.190 | 0.162 | 0.208 | 0.061 | 0.125 | 0.177 |
| **Sexual** | **27** | 0.176 | 0.120 | 0.085 | 0.166 | 0.174 | 0.230 | 0.292 | 0.226 | 0.280 | 0.254 | 0.188 | 0.247 | 0.362 | 0.185 | 0.293 | 0.350 |
|  | **28** | 0.057 | 0.075 | 0.077 | 0.113 | 0.121 | 0.169 | 0.177 | 0.158 | 0.170 | 0.128 | 0.103 | 0.193 | 0.143 | 0.046 | 0.194 | 0.325 |
|  | **29** | 0.076 | 0.065 | 0.064 | 0.185 | 0.206 | 0.141 | 0.208 | 0.212 | 0.222 | 0.182 | 0.141 | 0.149 | 0.255 | 0.100 | 0.172 | 0.264 |
| Spearman’s correlation coefficients, using pairwise deletion. Abbreviations: MENQOL, Menopause-Specific Quality of Life Questionnaire  Shading: r≥0.3, r≥0.5, N/A as same item. | | | | | | | | | | | | | | | | | |

**Table S3. Inter-item Spearman’s correlation coefficients for the MENQOL at Week 8**

| **Domain** | **Item** | **17** | **18** | **19** | **20** | **21** | **22** | **23** | **24** | **25** | **26** | **27** | **28** | **29** |
| --- | --- | --- | --- | --- | --- | --- | --- | --- | --- | --- | --- | --- | --- | --- |
| **VMS** | **1** | 0.145 | 0.193 | 0.114 | 0.120 | 0.076 | 0.124 | 0.138 | 0.116 | 0.128 | 0.043 | 0.176 | 0.057 | 0.076 |
|  | **2** | 0.161 | 0.187 | 0.092 | 0.108 | 0.064 | 0.051 | 0.113 | 0.148 | 0.138 | 0.054 | 0.120 | 0.075 | 0.065 |
|  | **3** | 0.164 | 0.158 | 0.108 | 0.110 | 0.077 | 0.098 | 0.158 | 0.115 | 0.119 | 0.065 | 0.085 | 0.077 | 0.064 |
| **Psychosocial** | **4** | 0.269 | 0.352 | 0.185 | 0.170 | 0.098 | 0.204 | 0.207 | 0.193 | 0.116 | 0.103 | 0.166 | 0.113 | 0.185 |
|  | **5** | 0.340 | 0.357 | 0.232 | 0.182 | 0.144 | 0.150 | 0.252 | 0.162 | 0.183 | 0.074 | 0.174 | 0.121 | 0.206 |
|  | **6** | 0.321 | 0.391 | 0.297 | 0.199 | 0.129 | 0.277 | 0.289 | 0.168 | 0.180 | 0.119 | 0.230 | 0.169 | 0.141 |
|  | **7** | 0.637 | 0.624 | 0.260 | 0.309 | 0.203 | 0.266 | 0.346 | 0.301 | 0.241 | 0.194 | 0.292 | 0.177 | 0.208 |
|  | **8** | 0.445 | 0.430 | 0.207 | 0.278 | 0.184 | 0.141 | 0.275 | 0.256 | 0.179 | 0.160 | 0.226 | 0.158 | 0.212 |
|  | **9** | 0.508 | 0.494 | 0.269 | 0.244 | 0.186 | 0.196 | 0.253 | 0.230 | 0.174 | 0.151 | 0.280 | 0.170 | 0.222 |
|  | **10** | 0.327 | 0.371 | 0.152 | 0.181 | 0.133 | 0.167 | 0.228 | 0.139 | 0.217 | 0.110 | 0.254 | 0.128 | 0.182 |
| **Physical** | **11** | 0.314 | 0.360 | 0.306 | 0.279 | 0.190 | 0.249 | 0.527 | 0.179 | 0.222 | 0.190 | 0.188 | 0.103 | 0.141 |
|  | **12** | 0.436 | 0.480 | 0.243 | 0.259 | 0.126 | 0.260 | 0.313 | 0.495 | 0.293 | 0.162 | 0.247 | 0.193 | 0.149 |
|  | **13** | 0.586 | 0.726 | 0.290 | 0.308 | 0.166 | 0.253 | 0.382 | 0.358 | 0.274 | 0.208 | 0.362 | 0.143 | 0.255 |
|  | **14** | 0.303 | 0.327 | 0.102 | 0.163 | 0.121 | 0.153 | 0.184 | 0.189 | 0.224 | 0.061 | 0.185 | 0.046 | 0.100 |
|  | **15** | 0.376 | 0.373 | 0.327 | 0.248 | 0.176 | 0.264 | 0.273 | 0.466 | 0.226 | 0.125 | 0.293 | 0.194 | 0.172 |
|  | **16** | 0.757 | 0.671 | 0.330 | 0.397 | 0.190 | 0.317 | 0.297 | 0.464 | 0.282 | 0.177 | 0.350 | 0.325 | 0.264 |
|  | **17** | 1.000 | 0.733 | 0.369 | 0.463 | 0.203 | 0.310 | 0.336 | 0.405 | 0.274 | 0.280 | 0.384 | 0.277 | 0.276 |
|  | **18** | 0.733 | 1.000 | 0.354 | 0.360 | 0.189 | 0.298 | 0.408 | 0.396 | 0.296 | 0.222 | 0.398 | 0.213 | 0.278 |
|  | **19** | 0.369 | 0.354 | 1.000 | 0.274 | 0.210 | 0.444 | 0.395 | 0.229 | 0.218 | 0.248 | 0.300 | 0.323 | 0.308 |
|  | **20** | 0.463 | 0.360 | 0.274 | 1.000 | 0.223 | 0.224 | 0.383 | 0.193 | 0.191 | 0.237 | 0.290 | 0.190 | 0.209 |
|  | **21** | 0.203 | 0.189 | 0.210 | 0.223 | 1.000 | 0.351 | 0.147 | 0.172 | 0.279 | 0.173 | 0.190 | 0.149 | 0.107 |
|  | **22** | 0.310 | 0.298 | 0.444 | 0.224 | 0.351 | 1.000 | 0.284 | 0.242 | 0.202 | 0.189 | 0.302 | 0.231 | 0.211 |
|  | **23** | 0.336 | 0.408 | 0.395 | 0.383 | 0.147 | 0.284 | 1.000 | 0.268 | 0.192 | 0.195 | 0.213 | 0.177 | 0.197 |
|  | **24** | 0.405 | 0.396 | 0.229 | 0.193 | 0.172 | 0.242 | 0.268 | 1.000 | 0.316 | 0.262 | 0.272 | 0.207 | 0.175 |
|  | **25** | 0.274 | 0.296 | 0.218 | 0.191 | 0.279 | 0.202 | 0.192 | 0.316 | 1.000 | 0.340 | 0.189 | 0.163 | 0.153 |
|  | **26** | 0.280 | 0.222 | 0.248 | 0.237 | 0.173 | 0.189 | 0.195 | 0.262 | 0.340 | 1.000 | 0.287 | 0.144 | 0.143 |
| **Sexual** | **27** | 0.384 | 0.398 | 0.300 | 0.290 | 0.190 | 0.302 | 0.213 | 0.272 | 0.189 | 0.287 | 1.000 | 0.467 | 0.681 |
|  | **28** | 0.277 | 0.213 | 0.323 | 0.190 | 0.149 | 0.231 | 0.177 | 0.207 | 0.163 | 0.144 | 0.467 | 1.000 | 0.496 |
|  | **29** | 0.276 | 0.278 | 0.308 | 0.209 | 0.107 | 0.211 | 0.197 | 0.175 | 0.153 | 0.143 | 0.681 | 0.496 | 1.000 |
| Spearman’s correlation coefficients, using pairwise deletion. Abbreviations: MENQOL, Menopause-Specific Quality of Life Questionnaire  Shading: r≥0.3, r≥0.5, N/A as same item. | | | | | | | | | | | | | | |

**Table S4. Graded response model parameter estimates for the PROMIS SD SF 8b T-score at Week 8**

| Item | Discrimination (SE) | Difficulty 1 (SE) | Difficulty 2 (SE) | Difficulty 3 (SE) | Difficulty 4 (SE) |
| --- | --- | --- | --- | --- | --- |
| 1: Restless | 2.82 (0.24) | -1.20 (0.10) | -0.16 (0.08) | 0.73 (0.08) | 1.71 (0.13) |
| 2: Satisfied | 3.90 (0.34) | -1.53 (0.11) | -0.52 (0.08) | 0.31 (0.07) | 1.02 (0.09) |
| 3: Refreshing | 3.11 (0.27) | -1.91 (0.14) | -0.72 (0.08) | 0.35 (0.07) | 1.14 (0.10) |
| 4: Difficulty falling asleep | 1.24 (0.14) | -0.61 (0.12) | 0.55 (0.12) | 1.50 (0.18) | 2.72 (0.30) |
| 5: Trouble staying asleep | 2.67 (0.23) | -1.85 (0.14) | -0.62 (0.09) | 0.28 (0.08) | 1.24 (0.11) |
| 6: Trouble sleeping | 3.40 (0.30) | -1.51 (0.11) | -0.46 (0.08) | 0.60 (0.08) | 1.58 (0.12) |
| 7: Get enough sleep | 2.76 (0.23) | -2.05 (0.16) | -0.52 (0.08) | 0.64 (0.08) | 1.72 (0.13) |
| 8: Sleep quality | 5.94 (0.69) | -1.67 (0.11) | -0.45 (0.07) | 0.64 (0.07) | 1.68 (0.11) |
| Abbreviations: PROMIS SD SF 8b, PROMIS Sleep Disturbance Short Form 8-item; SE, standard error | | | | | |

Table S5. Yen’s Q3 statistics for the PROMIS SD SF 8b T-score at Week 8

| Item | 1 | 2 | 3 | 4 | 5 | 6 | 7 | 8 |
| --- | --- | --- | --- | --- | --- | --- | --- | --- |
| 1: Restless | 1.000 | -0.176 | -0.322 | 0.218 | 0.154 | 0.161 | -0.297 | -0.304 |
| 2: Satisfied | -0.176 | 1.000 | 0.030 | -0.208 | -0.223 | -0.279 | -0.026 | -0.164 |
| 3: Refreshing | -0.322 | 0.030 | 1.000 | -0.164 | -0.273 | -0.350 | 0.030 | -0.089 |
| 4: Difficulty falling asleep | 0.218 | -0.208 | -0.164 | 1.000 | -0.049 | 0.238 | -0.072 | -0.187 |
| 5: Trouble staying asleep | 0.154 | -0.223 | -0.273 | -0.049 | 1.000 | 0.210 | -0.150 | -0.253 |
| 6: Trouble sleeping | 0.161 | -0.279 | -0.350 | 0.238 | 0.210 | 1.000 | -0.226 | -0.327 |
| 7: Get enough sleep | -0.297 | -0.026 | 0.030 | -0.072 | -0.150 | -0.226 | 1.000 | -0.053 |
| 8: Sleep quality | -0.304 | -0.164 | -0.089 | -0.187 | -0.253 | -0.327 | -0.053 | 1.000 |
| Abbreviations: PROMIS SD SF 8b, PROMIS Sleep Disturbance Short Form 8-item | | | | | | | | |

**Table S6. Known-groups differences at Week 8**

| **Known-groups** | **Groupings** | **n** | **Mean (SD)** | **Median** | **Min, Max** | **Between-group ES** | **F-test** | **Tukey vs. group 1** | **Tukey vs. group 2** | **Tukey vs. group 3** | **JT test** | **KW test** |
| --- | --- | --- | --- | --- | --- | --- | --- | --- | --- | --- | --- | --- |
| **HFDD Frequency score** | | | | | | | | | | | | |
| PGI-S Frequency | No HF [ref] | 25 | 0.10 (0.21) | 0.00 | 0.00, 0.86 |  | <.0001 |  |  |  | <.0001 | <.0001 |
|  | Rarely or Sometimes | 166 | 4.68 (7.00) | 2.57 | 0.00, 41.86 | 0.70 |  | 0.0105 |  |  |  |  |
|  | Often or Very often | 155 | 11.14 (8.16) | 9.29 | 0.43, 57.29 | 1.45 |  | <.0001 | <.0001 |  |  |  |
| **HFDD Severity score** | | | | | | | | | | | | |
| PGI-S Severity | No HF [ref] | 15 | 0.11 (0.20) | 0.00 | 0.00, 0.71 |  | <.0001 |  |  |  | <.0001 | <.0001 |
|  | Mild | 89 | 1.22 (0.58) | 1.22 | 0.00, 2.56 | 2.05 |  | <.0001 |  |  |  |  |
|  | Moderate | 124 | 1.84 (0.52) | 1.95 | 0.00, 3.00 | 3.48 |  | <.0001 | <.0001 |  |  |  |
|  | Severe or Very severe | 118 | 2.36 (0.49) | 2.40 | 0.00, 3.00 | 4.84 |  | <.0001 | <.0001 | <.0001 |  |  |
| **PROMIS SD SF 8b T-score** | | | | | | | | | | | | |
| PGI-S Sleep | No sleep problems [ref] | 41 | 42.74 (7.04) | 42.90 | 28.90, 61.50 |  | <.0001 |  |  |  | <.0001 | <.0001 |
|  | Mild | 105 | 47.77 (5.25) | 47.90 | 28.90, 58.30 | 0.87 |  | <.0001 |  |  |  |  |
|  | Moderate | 108 | 55.01 (4.49) | 55.30 | 39.80, 64.90 | 2.31 |  | <.0001 | <.0001 |  |  |  |
|  | Severe or Very severe | 95 | 62.52 (5.18) | 62.60 | 53.30, 76.50 | 3.41 |  | <.0001 | <.0001 | <.0001 |  |  |
| ISI Total Score | 0-7 (no clinically significant insomnia) [ref] | 121 | 45.09 (6.05) | 45.50 | 28.90, 58.30 |  | <.0001 |  |  |  | <.0001 | <.0001 |
|  | 8-14 (subthreshold insomnia) | 150 | 54.94 (4.98) | 55.30 | 38.00, 70.80 | 1.80 |  | <.0001 |  |  |  |  |
|  | 15-21 (moderate clinical insomnia) | 76 | 61.54 (4.27) | 61.50 | 53.30, 73.00 | 3.03 |  | <.0001 | <.0001 |  |  |  |
|  | 22-28 (severe clinical insomnia) | 12 | 68.04 (5.98) | 69.00 | 54.30, 76.50 | 3.80 |  | <.0001 | <.0001 | 0.0005 |  |  |
| **MENQOL Total score** | | | | | | | | | | | | |
| PGI-S Frequency | No HF [ref] | 25 | 2.19 (0.93) | 2.02 | 1.00, 4.20 |  | <.0001 |  |  |  | <.0001 | <.0001 |
|  | Rarely or Sometimes | 167 | 2.88 (1.10) | 2.75 | 1.00, 6.02 | 0.64 |  | 0.0102 |  |  |  |  |
|  | Often or Very often | 157 | 4.02 (1.12) | 3.86 | 1.63, 7.39 | 1.67 |  | <.0001 | <.0001 |  |  |  |
| PGI-S Severity | No HF [ref] | 15 | 1.95 (0.86) | 1.98 | 1.00, 4.20 |  | <.0001 |  |  |  | <.0001 | <.0001 |
|  | Mild | 90 | 2.62 (1.05) | 2.34 | 1.00, 5.34 | 0.65 |  | 0.1325 |  |  |  |  |
|  | Moderate | 125 | 3.32 (1.17) | 3.16 | 1.50, 7.39 | 1.19 |  | <.0001 | <.0001 |  |  |  |
|  | Severe or Very severe | 119 | 4.09 (1.08) | 3.96 | 1.42, 6.85 | 2.02 |  | <.0001 | <.0001 | <.0001 |  |  |

Abbreviations: HF, Hot Flash; HFDD, Hot Flash Daily Diary; ISI, Insomnia Severity Index; JT, Jonckheere-Terpstra; KW, Kruskal Wallis; MENQOL, Menopause-Specific Quality of Life Questionnaire; PGI-S, Patient Global Impression of Severity; PROMIS SD SF 8b, PROMIS Sleep Disturbance Short Form 8-item; SD, Standard Deviation

| **Table S7. Responsiveness correlations, change from baseline to Weeks 4 and 12** | | | | | | |
| --- | --- | --- | --- | --- | --- | --- |
| **Target instrument score** | **Convergent measure** | **Visit** | **Correlation coefficient** | **Hypothesis** | **n** | **Estimate** |
| **HFDD Frequency score** (change from Baseline) | PGI-S Frequency (change from Baseline) | Week 4 | Spearman's | >=0.4 | 336 | 0.526 |
|  |  | Week 12 | Spearman's | >=0.4 | 312 | 0.510 |
|  | PGI-C Frequency (absolute value at visit) | Week 4 | Polyserial | >=0.3 | 363 | 0.475 |
|  |  | Week 12 | Polyserial | >=0.3 | 339 | 0.330 |
| **HFDD Severity score** (change from Baseline) | PGI-S Severity (change from Baseline) | Week 4 | Spearman's | >=0.4 | 336 | 0.565 |
|  |  | Week 12 | Spearman's | >=0.4 | 312 | 0.598 |
|  | PGI-C Severity (absolute value at visit) | Week 4 | Polyserial | >=0.3 | 363 | 0.702 |
|  |  | Week 12 | Polyserial | >=0.3 | 339 | 0.685 |
| **PROMIS SD SF 8b T-score** (change from Baseline) | PGI-S Sleep (change from Baseline) | Week 4 | Spearman's | >=0.4 | 339 | 0.649 |
|  |  | Week 12 | Spearman's | >=0.4 | 313 | 0.638 |
|  | PGI-C Sleep (absolute value at visit) | Week 4 | Polyserial | >=0.3 | 352 | 0.698 |
|  |  | Week 12 | Polyserial | >=0.3 | 326 | 0.608 |
| **MENQOL Total score** (change from Baseline) | PGI-S Frequency (change from Baseline) | Week 4 | Spearman's | >=0.3 | 339 | 0.571 |
|  |  | Week 12 | Spearman's | >=0.3 | 313 | 0.541 |
|  | PGI-C Frequency (absolute value at visit) | Week 4 | Polyserial | >=0.3 | 348 | 0.561 |
|  |  | Week 12 | Polyserial | >=0.3 | 321 | 0.540 |
|  | PGI-S Severity (change from Baseline) | Week 4 | Spearman's | >=0.3 | 339 | 0.521 |
|  |  | Week 12 | Spearman's | >=0.3 | 313 | 0.498 |
|  | PGI-C Severity (absolute value at visit) | Week 4 | Polyserial | >=0.3 | 348 | 0.584 |
|  |  | Week 12 | Polyserial | >=0.3 | 321 | 0.515 |
| Abbreviations: FAS, Full Analysis Set; HF, hot flash; HFDD, Hot Flash Daily Diary; PGI-C, Patient Global Impression of Change; PGI-S, Patient Global Impression of Severity; PROMIS SD SF 8b, PROMIS Sleep Disturbance Short Form 8-item; MENQOL, Menopause-specific Quality of Life Questionnaire PGI-S is change from Baseline to the specified visit, PGI-C is score at the visit. | | | | | | |

**Score interpretation: Anchor-based descriptive statistics**

Decreases in HFDD Frequency and HFDD Severity scores (mean and median) at Week 4 and Week 12 (Table S8 to Table S11) were observed in stable, ‘minimal improvement’ and ‘much improvement’ groups. For both HFDD scores and across all anchors, the greatest amount of change (i.e., decrease) was observed in the ‘much improvement’ group, followed by the ‘minimal improvement’ group, with the least amount of change observed in the ‘stable’ group. Sample sizes for ‘improved’ and ‘stable’ groups across all analyses were considered sufficient and so no anchor categories were collapsed.

Table S8. Anchor-based descriptive statistics for HFDD Frequency, change from baseline to Week 4

| Anchor measure | Anchor category | n | Mean (SD) | 95% CI | Min | 10th percentile | 25th percentile | Median | 75th percentile | 90th percentile | Max |
| --- | --- | --- | --- | --- | --- | --- | --- | --- | --- | --- | --- |
| PGI-S Frequency | Much improvement: change from baseline =-2 | 79 | -10.09 (8.08) | -11.90, -8.28 | -53.14 | -18.86 | -14.64 | -8.21 | -5.79 | -2.57 | 11.57 |
|  | Minimal improvement: change from baseline =-1 | 114 | -6.73 (8.77) | -8.36, -5.11 | -72.21 | -14.36 | -8.21 | -5.50 | -2.21 | -0.14 | 6.57 |
|  | Stable: change from baseline =0 | 101 | -3.34 (5.71) | -4.47, -2.22 | -31.31 | -8.71 | -5.43 | -2.36 | -0.29 | 1.36 | 10.93 |
| PGI-C Frequency | Much improvement: Much less | 116 | -12.08 (9.13) | -13.76, -10.40 | -75.71 | -19.86 | -14.68 | -9.64 | -6.86 | -5.60 | -1.21 |
|  | Minimal improvement: A little less | 132 | -7.26 (9.65) | -8.92, -5.60 | -72.21 | -14.07 | -8.43 | -5.29 | -3.29 | -0.71 | 7.64 |
|  | Stable: The same (no change) | 92 | -2.45 (4.47) | -3.38, -1.53 | -20.71 | -7.64 | -4.68 | -1.54 | 0.00 | 1.36 | 10.93 |
| Abbreviations: CI, Confidence Interval; FAS, Full Analysis Set; HF, hot flash; HFDD, Hot Flash Daily Diary; PGI-C, Patient Global Impression of Change; PGI-S, Patient Global Impression of Severity; SD, Standard Deviation | | | | | | | | | | | |

Table S9. Anchor-based descriptive statistics for HFDD Frequency, change from baseline to Week 12

| Anchor measure | Anchor category | n | Mean (SD) | 95% CI | Min | 10th percentile | 25th percentile | Median | 75th percentile | 90th percentile | Max |
| --- | --- | --- | --- | --- | --- | --- | --- | --- | --- | --- | --- |
| PGI-S Frequency | Much improvement: change from baseline =-2 | 86 | -10.54 (8.08) | -12.27, -8.81 | -52.43 | -18.36 | -12.07 | -8.96 | -5.93 | -3.86 | 0.64 |
|  | Minimal improvement: change from baseline =-1 | 101 | -7.72 (9.35) | -9.57, -5.87 | -75.64 | -13.64 | -9.50 | -6.29 | -3.50 | -0.64 | 6.43 |
|  | Stable: change from baseline =0 | 74 | -4.40 (9.14) | -6.52, -2.28 | -62.71 | -10.21 | -6.29 | -2.64 | -0.29 | 1.79 | 15.00 |
|  |  |  |  |  |  |  |  |  |  |  |  |
| PGI-C Frequency | Much improvement: Much less | 136 | -12.14 (8.45) | -13.57, -10.71 | -75.64 | -20.14 | -15.18 | -9.86 | -7.64 | -5.79 | -3.86 |
|  | Minimal improvement: A little less | 103 | -7.49 (9.38) | -9.32, -5.65 | -76.14 | -12.21 | -9.50 | -6.29 | -3.36 | -0.64 | 3.79 |
|  | Stable: The same (no change) | 83 | -3.59 (6.24) | -4.95, -2.23 | -29.74 | -9.36 | -6.07 | -2.43 | -0.21 | 1.64 | 15.00 |
| Abbreviations: CI, Confidence Interval; FAS, Full Analysis Set; HF, hot flash; HFDD, Hot Flash Daily Diary; PGI-C, Patient Global Impression of Change; PGI-S, Patient Global Impression of Severity; SD, Standard Deviation | | | | | | | | | | | |

Table S10. Anchor-based descriptive statistics for HFDD Severity, change from baseline to Week 4

| Anchor measure | Anchor category | n | Mean (SD) | 95% CI | Min | 10th percentile | 25th percentile | Median | 75th percentile | 90th percentile | Max |
| --- | --- | --- | --- | --- | --- | --- | --- | --- | --- | --- | --- |
| PGI-S Severity | Much improvement: change from baseline =-2 | 75 | -1.04 (0.59) | -1.18, -0.91 | -2.63 | -1.92 | -1.28 | -1.00 | -0.68 | -0.32 | 0.00 |
|  | Minimal improvement: change from baseline =-1 | 116 | -0.54 (0.46) | -0.63, -0.46 | -2.06 | -1.21 | -0.75 | -0.49 | -0.25 | 0.00 | 0.57 |
|  | Stable: change from baseline =0 | 105 | -0.33 (0.38) | -0.40, -0.26 | -1.84 | -0.79 | -0.52 | -0.26 | -0.08 | 0.07 | 0.38 |
| PGI-C Severity | Much improvement: Much less | 104 | -1.25 (0.70) | -1.38, -1.11 | -2.77 | -2.22 | -1.75 | -1.20 | -0.76 | -0.43 | 0.38 |
|  | Minimal improvement: A little less | 134 | -0.52 (0.40) | -0.59, -0.45 | -1.97 | -1.04 | -0.76 | -0.50 | -0.23 | -0.05 | 0.68 |
|  | Stable: The same (no change) | 109 | -0.27 (0.30) | -0.32, -0.21 | -1.19 | -0.64 | -0.49 | -0.24 | -0.01 | 0.07 | 0.57 |
| Abbreviations: CI, Confidence Interval; FAS, Full Analysis Set; HF, hot flash; HFDD, Hot Flash Daily Diary; PGI-C, Patient Global Impression of Change; PGI-S, Patient Global Impression of Severity; SD, Standard Deviation  Source: **: Table 5.1 / 3** | | | | | | | | | | | |

Table S11. Anchor-based descriptive statistics for HFDD Severity, change from baseline to Week 12

| Anchor measure | Anchor category | n | Mean (SD) | 95% CI | Min | 10^th^ percentile | 25^th^ percentile | Median | 75^th^ percentile | 90^th^ percentile | Max |
| --- | --- | --- | --- | --- | --- | --- | --- | --- | --- | --- | --- |
| PGI-S Severity | Much improvement: change from baseline =-2 | 74 | -1.22 (0.68) | -1.38, -1.07 | -2.90 | -2.26 | -1.62 | -1.19 | -0.65 | -0.42 | -0.10 |
|  | Minimal improvement: change from baseline =-1 | 128 | -0.67 (0.59) | -0.77, -0.57 | -2.43 | -1.51 | -0.97 | -0.52 | -0.25 | 0.00 | 0.57 |
|  | Stable: change from baseline =0 | 69 | -0.33 (0.39) | -0.42, -0.23 | -1.45 | -0.77 | -0.60 | -0.28 | -0.03 | 0.06 | 0.59 |
| PGI-C Severity | Much improvement: Much less | 129 | -1.39 (0.77) | -1.53, -1.26 | -2.90 | -2.42 | -2.05 | -1.31 | -0.85 | -0.38 | 0.57 |
|  | Minimal improvement: A little less | 107 | -0.58 (0.45) | -0.67, -0.50 | -2.43 | -1.15 | -0.87 | -0.55 | -0.31 | -0.05 | 0.59 |
|  | Stable: The same (no change) | 87 | -0.29 (0.37) | -0.37, -0.21 | -1.45 | -0.80 | -0.48 | -0.24 | -0.02 | 0.09 | 0.30 |
| Abbreviations: CI, Confidence Interval; FAS, Full Analysis Set; HF, hot flash; HFDD, Hot Flash Daily Diary; PGI-C, Patient Global Impression of Change; PGI-S, Patient Global Impression of Severity; SD, Standard Deviation | | | | | | | | | | | |

All estimates monotonically decreased in the expected direction from baseline to Week 4 and baseline to Week 12, suggesting that both the PGI-S Sleep and PGI-C Sleep are appropriate anchors for the PROMIS SD SF 8b T-score (Table S12 and S13). Sample sizes within ‘improved’ and ‘stable’ groups were deemed sufficient to draw conclusions.

Table S12. Anchor-based descriptive statistics for PROMIS SD SF 8b T-score, change from baseline to Week 4

| Anchor measure | Anchor category | n | Mean (SD) | 95% CI | Min | 10th percentile | 25th percentile | Median | 75th percentile | 90th percentile | Max |
| --- | --- | --- | --- | --- | --- | --- | --- | --- | --- | --- | --- |
| PGI-S Sleep | Much improvement: change from baseline =-2 | 68 | -11.97 (6.15) | -13.46, -10.48 | -24.30 | -20.30 | -16.30 | -12.35 | -8.45 | -2.30 | 1.20 |
|  | Minimal improvement: change from baseline =-1 | 121 | -6.74 (5.27) | -7.69, -5.79 | -19.60 | -13.60 | -9.80 | -6.40 | -3.80 | 0.00 | 18.70 |
|  | Stable: change from baseline =0 | 97 | -2.34 (5.42) | -3.43, -1.25 | -19.30 | -8.60 | -6.10 | -1.20 | 1.40 | 4.50 | 9.20 |
| PGI-C Sleep | Much improvement: Much less | 85 | -15.22 (7.38) | -16.81, -13.62 | -32.60 | -24.80 | -19.60 | -15.20 | -10.40 | -5.70 | 0.00 |
|  | Minimal improvement: A little less | 103 | -7.93 (5.25) | -8.96, -6.91 | -22.20 | -14.80 | -11.10 | -7.50 | -5.10 | 0.00 | 5.10 |
|  | Stable: The same (no change) | 146 | -3.01 (5.63) | -3.93, -2.09 | -19.30 | -10.60 | -6.60 | -2.40 | 0.00 | 4.10 | 18.70 |
| Abbreviations: CI, Confidence Interval; FAS, Full Analysis Set; PGI-C, Patient Global Impression of Change; PGI-S, Patient Global Impression of Severity; PROMIS SD SF 8b, PROMIS Sleep Disturbance Short Form 8-item; SD, Standard Deviation | | | | | | | | | | | |

Table S13. Anchor-based descriptive statistics for PROMIS SD SF 8b T-score, change from baseline to Week 12

| Anchor measure | Anchor category | n | Mean (SD) | 95% CI | Min | 10th percentile | 25th percentile | Median | 75th percentile | 90th percentile | Max |
| --- | --- | --- | --- | --- | --- | --- | --- | --- | --- | --- | --- |
| PGI-S Sleep | Much improvement: change from baseline =-2 | 79 | -12.82 (6.33) | -14.24, -11.40 | -29.50 | -20.60 | -16.50 | -12.80 | -9.20 | -4.10 | 4.00 |
|  | Minimal improvement: change from baseline =-1 | 111 | -6.78 (5.50) | -7.81, -5.75 | -19.20 | -14.80 | -10.40 | -6.20 | -2.30 | 0.00 | 4.20 |
|  | Stable: change from baseline =0 | 81 | -3.00 (4.69) | -4.04, -1.96 | -14.80 | -9.20 | -5.50 | -3.10 | 0.00 | 2.80 | 7.10 |
| PGI-C Sleep | Much improvement: Much less | 89 | -14.95 (6.39) | -16.30, -13.61 | -31.50 | -25.10 | -17.30 | -14.10 | -10.80 | -6.70 | 1.00 |
|  | Minimal improvement: A little less | 79 | -7.62 (7.23) | -9.24, -6.01 | -29.80 | -18.20 | -11.70 | -7.20 | -2.10 | 1.00 | 7.20 |
|  | Stable: The same (no change) | 128 | -4.78 (5.87) | -5.81, -3.76 | -36.70 | -11.60 | -7.55 | -4.10 | -1.20 | 2.00 | 10.40 |
| Abbreviations: CI, Confidence Interval; FAS, Full Analysis Set; PGI-C, Patient Global Impression of Change; PGI-S, Patient Global Impression of Severity; PROMIS SD SF 8b, PROMIS Sleep Disturbance Short Form 8-item; SD, Standard Deviation | | | | | | | | | | | |

All estimates monotonically decreased in the expected direction from baseline to Week 4 and baseline to Week 12, suggesting that both the PGI-S Frequency, PGI-C Frequency, PGI-S Severity and PGI-C Severity are appropriate anchors for the MENQOL scores (Table S14 and Table S15). Sample sizes in the ‘improved’ and ‘stable’ groups were deemed sufficient to draw conclusions.

Table S14. Anchor-based descriptive statistics for MENQOL Total score, change from baseline to Week 4

| Anchor measure | Anchor category | n | Mean (SD) | 95% CI | Min | 10th percentile | 25th percentile | Median | 75th percentile | 90th percentile | Max |
| --- | --- | --- | --- | --- | --- | --- | --- | --- | --- | --- | --- |
| PGI-S Frequency | Much improvement: change from baseline =-2 | 79 | -1.65 (1.03) | -1.88, -1.42 | -4.93 | -2.93 | -2.42 | -1.55 | -0.93 | -0.29 | 0.81 |
|  | Minimal improvement: change from baseline =-1 | 115 | -0.75 (0.99) | -0.93, -0.56 | -4.33 | -1.92 | -1.25 | -0.66 | -0.12 | 0.35 | 2.23 |
|  | Stable: change from baseline =0 | 101 | -0.32 (0.89) | -0.49, -0.14 | -3.41 | -1.22 | -0.84 | -0.26 | 0.21 | 0.77 | 1.71 |
| PGI-C Frequency | Much improvement: Much less | 113 | -1.92 (1.20) | -2.14, -1.69 | -5.01 | -3.52 | -2.72 | -1.82 | -0.91 | -0.63 | 0.55 |
|  | Minimal improvement: A little less | 122 | -0.83 (1.09) | -1.03, -0.64 | -4.38 | -2.10 | -1.47 | -0.69 | -0.10 | 0.41 | 2.23 |
|  | Stable: The same (no change) | 92 | -0.34 (0.75) | -0.49, -0.18 | -2.98 | -1.19 | -0.93 | -0.26 | 0.16 | 0.59 | 1.25 |
| PGI-S Severity | Much improvement: change from baseline =-2 | 77 | -1.69 (1.31) | -1.99, -1.40 | -4.93 | -3.69 | -2.55 | -1.43 | -0.77 | -0.29 | 1.12 |
|  | Minimal improvement: change from baseline =-1 | 116 | -0.97 (0.88) | -1.13, -0.81 | -4.38 | -1.92 | -1.49 | -0.91 | -0.31 | 0.00 | 1.17 |
|  | Stable: change from baseline =0 | 106 | -0.38 (0.95) | -0.56, -0.19 | -2.83 | -1.81 | -0.84 | -0.22 | 0.19 | 0.71 | 2.23 |
| PGI-C Severity | Much improvement: Much less | 100 | -2.01 (1.23) | -2.25, -1.76 | -5.01 | -3.66 | -2.76 | -1.90 | -1.05 | -0.69 | 0.81 |
|  | Minimal improvement: A little less | 126 | -0.94 (1.08) | -1.13, -0.75 | -4.38 | -2.38 | -1.56 | -0.86 | -0.23 | 0.23 | 2.23 |
|  | Stable: The same (no change) | 108 | -0.29 (0.75) | -0.43, -0.15 | -1.98 | -1.19 | -0.86 | -0.26 | 0.18 | 0.68 | 1.71 |
| Abbreviations: CI, Confidence Interval; FAS, Full Analysis Set; MENQOL, Menopause-specific Quality of Life Questionnaire; PGI-C, Patient Global Impression of Change; PGI-S, Patient Global Impression of Severity; SD, Standard Deviation | | | | | | | | | | | |

Table S15. Anchor-based descriptive statistics for MENQOL Total score, change from baseline to Week 12

| Anchor measure | Anchor category | n | Mean (SD) | 95% CI | Min | 10th percentile | 25th percentile | Median | 75th percentile | 90th percentile | Max |
| --- | --- | --- | --- | --- | --- | --- | --- | --- | --- | --- | --- |
| PGI-S Frequency | Much improvement: change from baseline =-2 | 86 | -1.55 (1.16) | -1.79, -1.30 | -5.52 | -2.74 | -2.15 | -1.41 | -0.83 | -0.26 | 0.92 |
|  | Minimal improvement: change from baseline =-1 | 101 | -0.94 (1.08) | -1.15, -0.72 | -4.41 | -2.22 | -1.63 | -0.77 | -0.24 | 0.40 | 2.03 |
|  | Stable: change from baseline =0 | 75 | -0.39 (0.86) | -0.58, -0.19 | -2.45 | -1.68 | -0.84 | -0.33 | 0.14 | 0.53 | 2.27 |
| PGI-C Frequency | Much improvement: Much less | 127 | -1.92 (1.17) | -2.13, -1.72 | -5.76 | -3.49 | -2.45 | -1.79 | -1.12 | -0.58 | 0.39 |
|  | Minimal improvement: A little less | 96 | -0.73 (1.15) | -0.96, -0.50 | -4.50 | -2.27 | -1.51 | -0.66 | -0.01 | 0.53 | 2.27 |
|  | Stable: The same (no change) | 81 | -0.56 (0.78) | -0.74, -0.39 | -2.44 | -1.69 | -1.05 | -0.46 | -0.09 | 0.45 | 1.10 |
| PGI-S Severity | Much improvement: change from baseline =-2 | 74 | -1.79 (1.20) | -2.07, -1.52 | -5.52 | -3.08 | -2.34 | -1.77 | -1.12 | -0.32 | 1.33 |
|  | Minimal improvement: change from baseline =-1 | 129 | -0.94 (1.07) | -1.13, -0.76 | -4.50 | -2.33 | -1.62 | -0.83 | -0.26 | 0.44 | 2.03 |
|  | Stable: change from baseline =0 | 69 | -0.53 (0.77) | -0.71, -0.34 | -2.45 | -1.69 | -1.07 | -0.44 | -0.01 | 0.41 | 1.09 |
| PGI-C Severity | Much improvement: Much less | 120 | -1.89 (1.24) | -2.11, -1.66 | -5.76 | -3.45 | -2.43 | -1.83 | -1.14 | -0.37 | 1.66 |
|  | Minimal improvement: A little less | 100 | -0.86 (1.18) | -1.09, -0.62 | -4.50 | -2.36 | -1.68 | -0.73 | -0.06 | 0.51 | 2.27 |
|  | Stable: The same (no change) | 85 | -0.60 (0.74) | -0.76, -0.44 | -2.44 | -1.68 | -1.10 | -0.57 | -0.17 | 0.40 | 1.10 |
| Abbreviations: CI, Confidence Interval; FAS, Full Analysis Set; MENQOL, Menopause-specific Quality of Life Questionnaire; PGI-C, Patient Global Impression of Change; PGI-S, Patient Global Impression of Severity; SD, Standard Deviation | | | | | | | | | | | |

**Score interpretation: Triangulation at Week 4**

**Table S16. Triangulation of anchor-based within-individual thresholds at Week 4**

| **Magnitude** | **Method** | **Anchor** | **Estimate (95% CI)** | **z-transformed correlation** | **Weighted average (95% CI)** |
| --- | --- | --- | --- | --- | --- |
| **HFDD Frequency score** | | | | | |
| Minimal | Logistic regression | PGI-S Frequency | -4.68 (-5.56; -3.92) | 0.58 | -4.96 (-5.66; -4.26) |
|  |  | PGI-C Frequency | -4.19 (-4.98; -3.51) | 0.52 |  |
|  | Discriminant analysis | PGI-S Frequency | -5.87 (-6.97; -4.75) | 0.58 |  |
|  |  | PGI-C Frequency | -5.01 (-6.09; -4.09) | 0.52 |  |
| Much | Logistic regression | PGI-S Frequency | -8.12 (-9.11; -7.28) | 0.58 | -7.51 (-8.35; -6.67) |
|  |  | PGI-C Frequency | -8.38 (-9.44; -7.61) | 0.52 |  |
|  | Discriminant analysis | PGI-S Frequency | -7.00 (-8.45; -5.51) | 0.58 |  |
|  |  | PGI-C Frequency | -6.53 (-7.91; -5.18) | 0.52 |  |
| **HFDD Severity score** | | | | | |
| Minimal | Logistic regression | PGI-S Severity | -0.48 (-0.54; -0.43) | 0.64 | -0.55 (-0.65; -0.44) |
|  |  | PGI-C Severity | -0.44 (-0.49; -0.39) | 0.87 |  |
|  | Discriminant analysis | PGI-S Severity | -0.63 (-0.73; -0.50) | 0.64 |  |
|  |  | PGI-C Severity | -0.64 (-0.74; -0.53) | 0.87 |  |
| Much | Logistic regression | PGI-S Severity | -0.82 (-0.90; -0.75) | 0.64 | -0.80 (-0.83; -0.76) |
|  |  | PGI-C Severity | -0.83 (-0.90; -0.76) | 0.87 |  |
|  | Discriminant analysis | PGI-S Severity | -0.74 (-0.82; -0.66) | 0.64 |  |
|  |  | PGI-C Severity | -0.79 (-0.89; -0.70) | 0.87 |  |
| **PROMIS SD SF 8b T-score** | | | | | |
| Minimal | Logistic regression | PGI-S Sleep | -5.00 (-5.78; -4.24) | 0.77 | -6.09 (-6.88; -5.30) |
|  |  | PGI-C Sleep | -6.60 (-7.25; -5.95) | 0.86 |  |
|  | Discriminant analysis | PGI-S Sleep | -5.85 (-7.96; -3.70) | 0.77 |  |
|  |  | PGI-C Sleep | -6.79 (-8.07; -5.56) | 0.86 |  |
| Much | Logistic regression | PGI-S Sleep | -9.97 (-10.82; -9.16) | 0.77 | -10.12 (-10.73; -9.51) |
|  |  | PGI-C Sleep | -10.96 (-11.90; -10.09) | 0.86 |  |
|  | Discriminant analysis | PGI-S Sleep | -9.53 (-10.76; -8.34) | 0.77 |  |
|  |  | PGI-C Sleep | -9.93 (-11.02; -8.85) | 0.86 |  |
| **MENQOL Total score** | | | | | |
| Minimal | Logistic regression | PGI-S Frequency | -0.64 (-0.77; -0.52) | 0.65 | -0.82 (-0.95; -0.69) |
|  |  | PGI-C Frequency | -0.67 (-0.79; -0.55) | 0.63 |  |
|  |  | PGI-S Severity | -0.74 (-0.86; -0.60) | 0.58 |  |
|  |  | PGI-C Severity | -0.69 (-0.80; -0.58) | 0.67 |  |
|  | Discriminant analysis | PGI-S Frequency | -0.89 (-1.17; -0.66) | 0.65 |  |
|  |  | PGI-C Frequency | -1.11 (-1.33; -0.75) | 0.63 |  |
|  |  | PGI-S Severity | -0.73 (-0.95; -0.53) | 0.58 |  |
|  |  | PGI-C Severity | -1.07 (-1.33; -0.76) | 0.67 |  |
| Much | Logistic regression | PGI-S Frequency | -1.26 (-1.39; -1.14) | 0.65 | -1.27 (-1.35; -1.19) |
|  |  | PGI-C Frequency | -1.31 (-1.46; -1.18) | 0.63 |  |
|  |  | PGI-S Severity | -1.38 (-1.54; -1.23) | 0.58 |  |
|  |  | PGI-C Severity | -1.40 (-1.55; -1.26) | 0.67 |  |
|  | Discriminant analysis | PGI-S Frequency | -1.09 (-1.31; -0.89) | 0.65 |  |
|  |  | PGI-C Frequency | -1.15 (-1.48; -0.84) | 0.63 |  |
|  |  | PGI-S Severity | -1.35 (-1.78; -0.94) | 0.58 |  |
|  |  | PGI-C Severity | -1.22 (-1.51; -0.95) | 0.67 |  |
| Abbreviations: CI, Confidence Interval; FAS, Full Analysis Set; HF, hot flash; HFDD, Hot Flash Daily Diary; MENQOL, Menopause-specific Quality of Life Questionnaire; PROMIS SD SF 8b, PROMIS Sleep Disturbance Short Form 8-item; PGI-C, Patient Global Impression of Change; PGI-S, Patient Global Impression of Severity.  Only anchor instruments correlated >=0.3 with the PRO score of interest are used to obtain estimates here. If correlation is <0.3, estimate is labeled NA (not applicable). Weighted average only calculated if >1 suitable estimate available. | | | | | |

**Score interpretation: CDF plots**

The CDF plots for the HFDD Frequency (Week 12), PROMIS SD SF 8b T-score (Week 12) and MENQOL Total score (Week 12) are presented below. The CDF plots for these scores showed clear and consistent separation of groups; as such, the PDF plots are not presented.

For the HFDD Frequency change from baseline to Week 12, CDF curves support the ‘minimally important’ adjusted thresholds for PGI-S Frequency of -5.45 produced by logistic regression and of -5.25 for discriminant analysis (Figure S1). Both thresholds appear to correctly classify the majority of ‘improved’ participants, while correctly excluding the majority of stable participants. CDF curves support the ‘minimally important’ adjusted thresholds for PGI-C Frequency of -5.76 produced by logistic regression and of -5.29 for discriminant analysis (Figure S2). Both thresholds appear to correctly classify the majority of ‘improved’ participants, while correctly excluding the majority of stable participants.

Figure S1. CDF for HFDD Frequency, change from baseline to Week 12


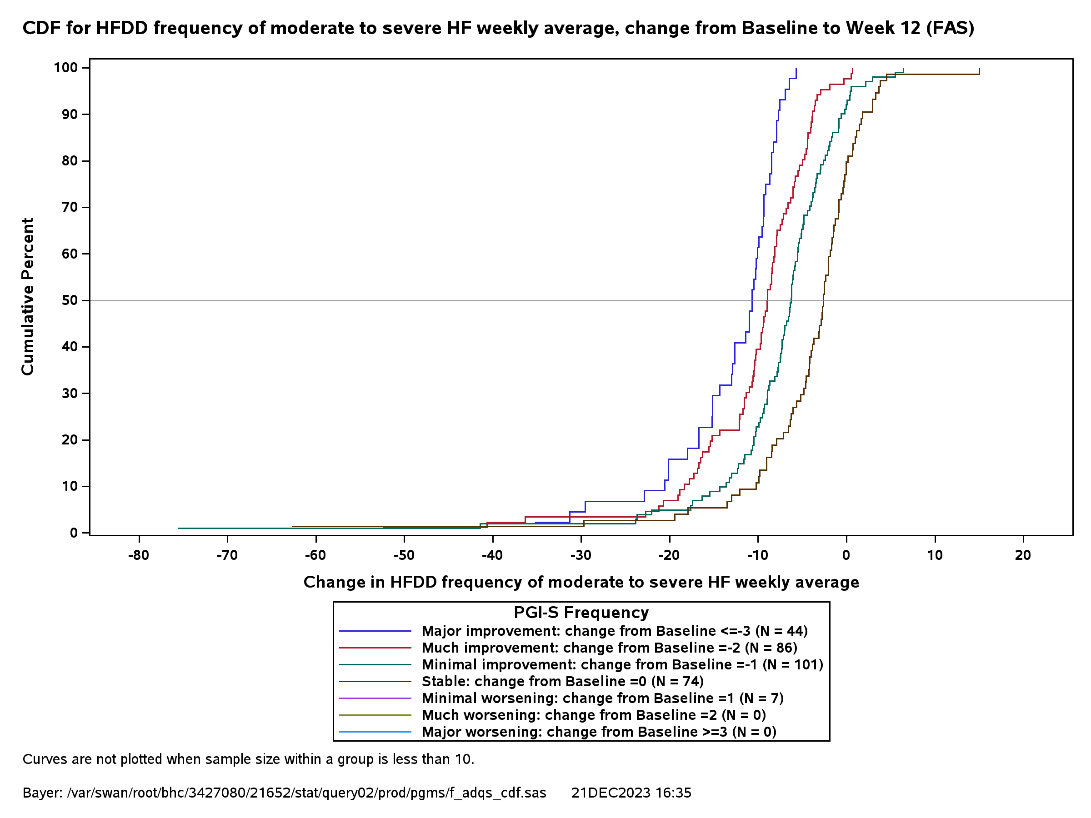


Figure S2. CDF for HFDD Frequency, change from baseline to Week 12


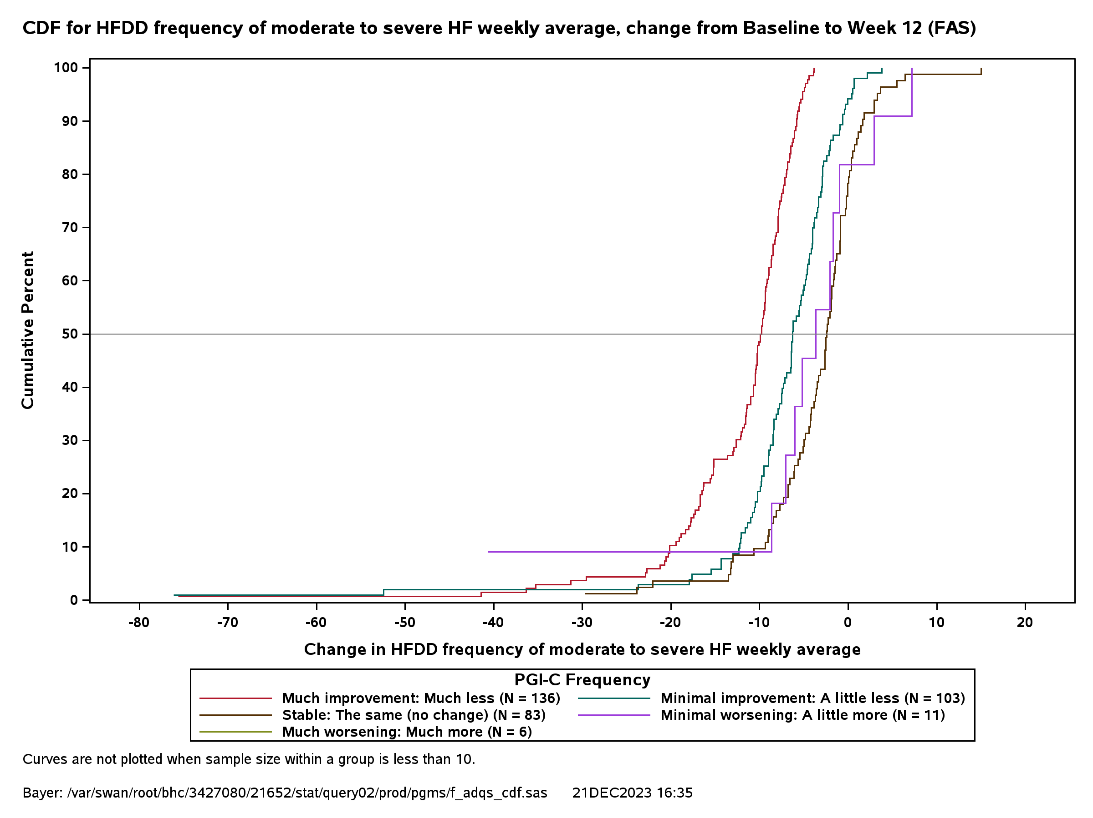


For the PROMIS SD SF 8b change from baseline to Week 12, CDF curves support the ‘minimally important’ adjusted thresholds for PGI-S Sleep of -5.20 produced by logistic regression and of -6.96 for discriminant analysis (Figure S3). Both thresholds appear to correctly classify the majority of ‘improved’ participants, while correctly excluding the majority of stable participants. CDF curves support the ‘minimally important’ adjusted thresholds for PGI-C Sleep of -7.53 produced by logistic regression and of -8.34 for discriminant analysis (Figure S4). Both thresholds appear to correctly classify the majority of ‘improved’ participants, while correctly excluding the majority of stable participants.

Figure S3. CDF for PROMIS SD SF 8b T-score, change from baseline to Week 12


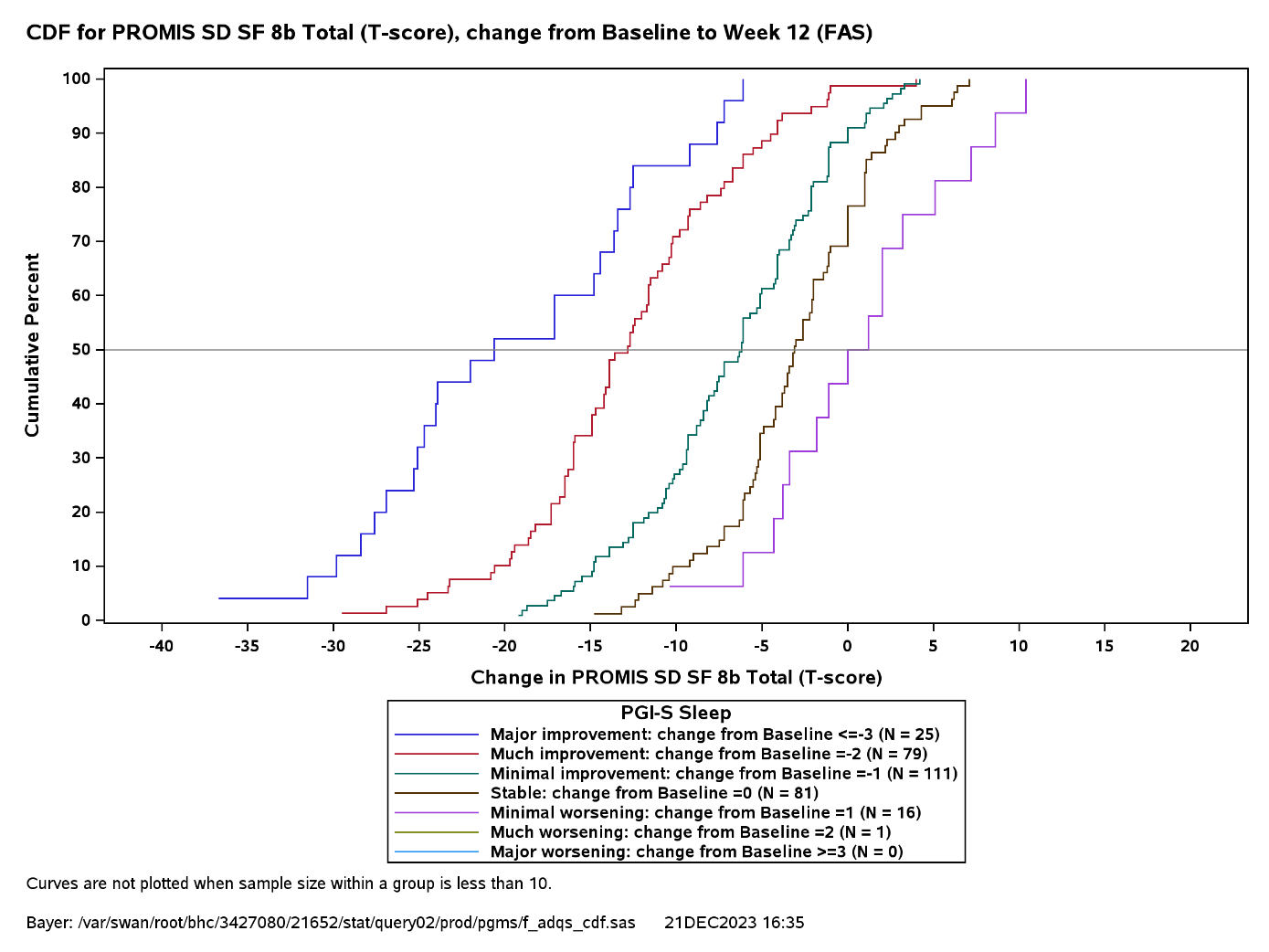


Figure S4. CDF for PROMIS SD SF 8b T-score, change from baseline to Week 12


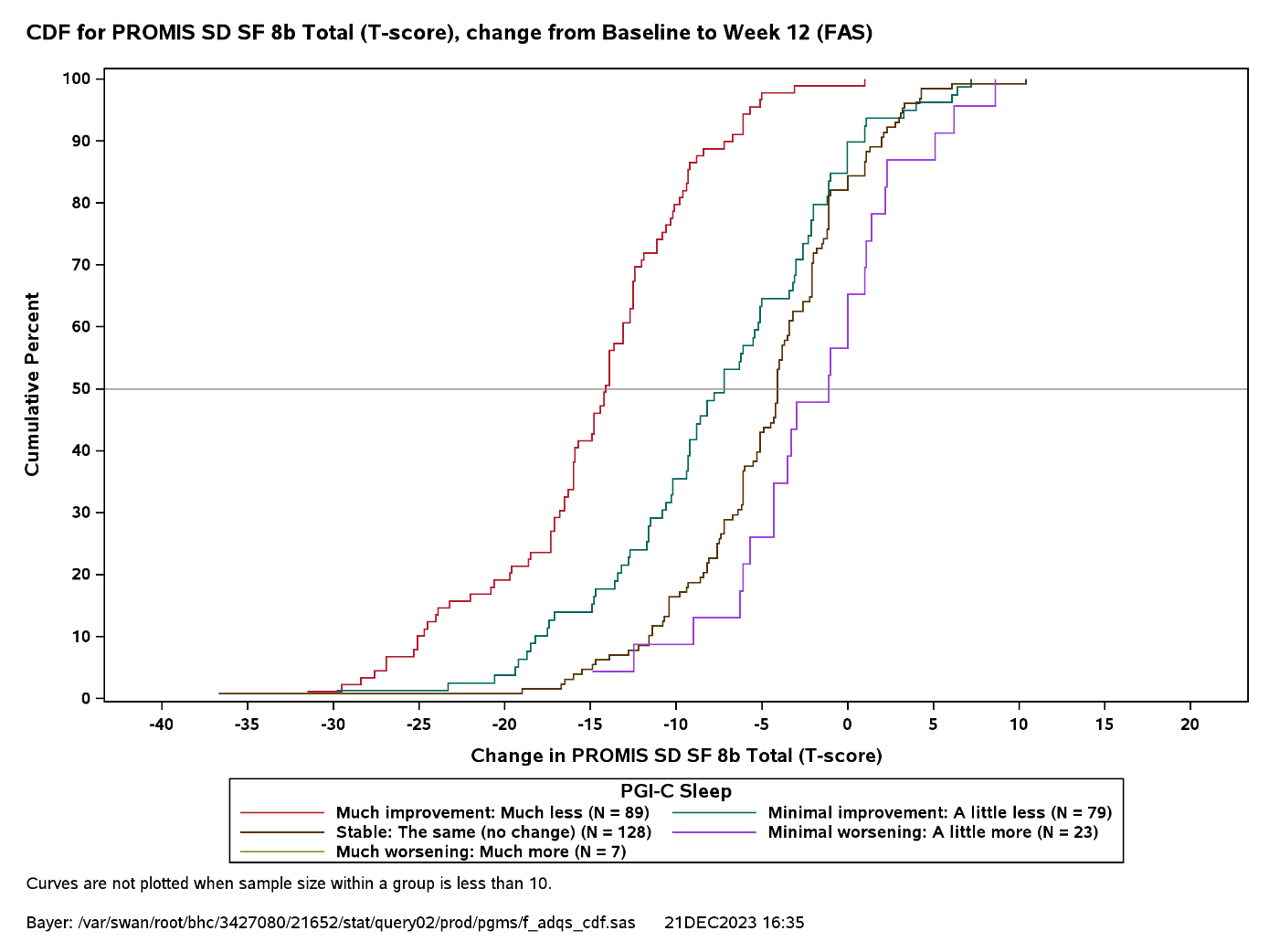


For the MENQOL Total score change from baseline to Week 12, CDF curves support the ‘minimally important’ adjusted thresholds for PGI-S Frequency of -0.68 produced by logistic regression and of -0.92 for discriminant analysis (Figure S5). Both thresholds appear to correctly classify the majority of ‘improved’ participants, while correctly excluding the majority of stable participants. CDF curves support the ‘minimally important’ adjusted thresholds for PGI-C Frequency of -0.82 produced by logistic regression and of -1.12 for discriminant analysis (Figure S6). Both thresholds appear to correctly classify the majority of ‘improved’ participants, while correctly excluding the majority of stable participants. However, there was overlap between curves of the ‘minimal improvement’ and ‘stable’ subjects according to the PGI-C Frequency anchor.

Figure S5. CDF for MENQOL Total score, change from baseline to Week 12


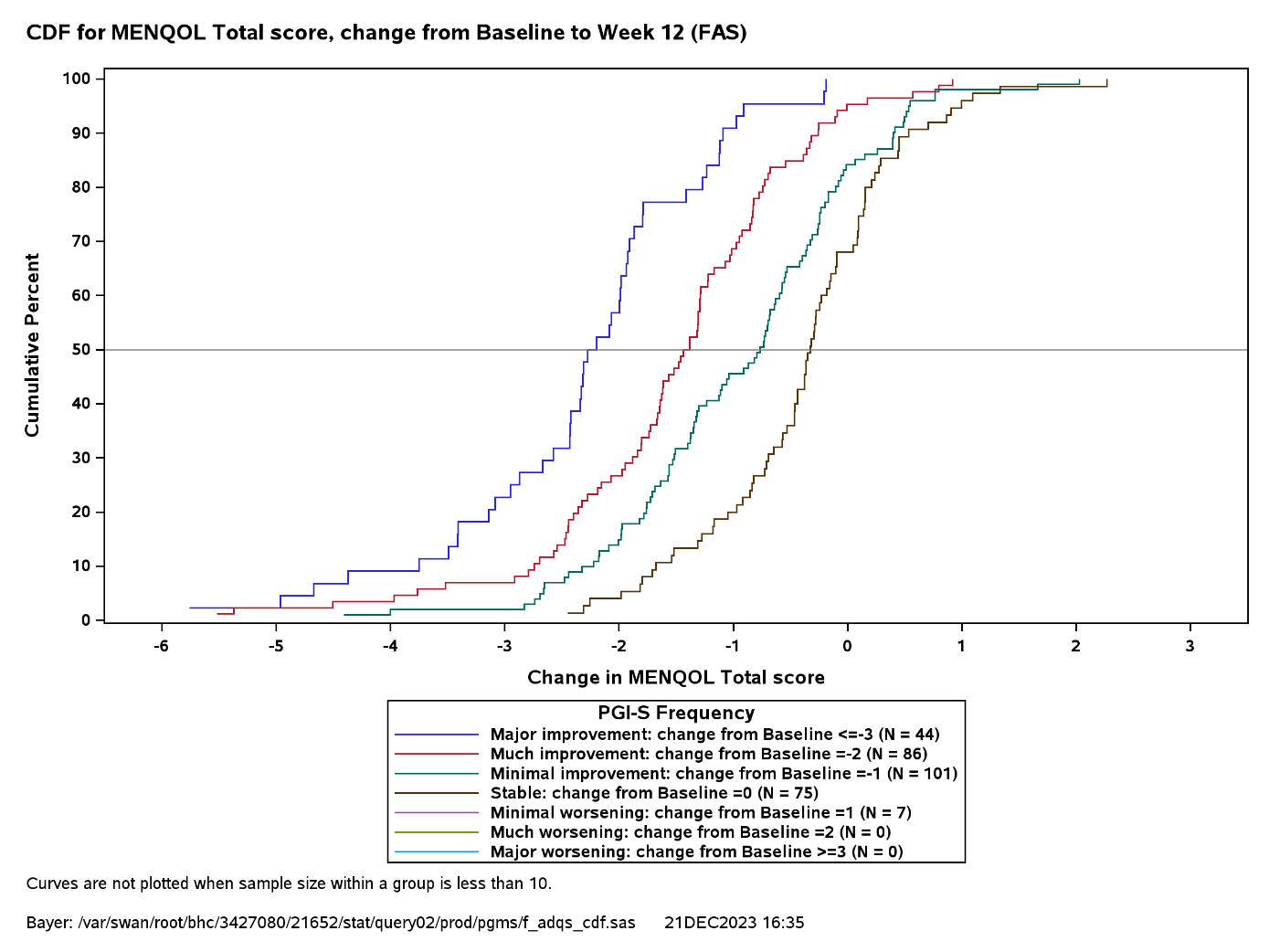


Figure S6. CDF for MENQOL Total score, change from baseline to Week 12


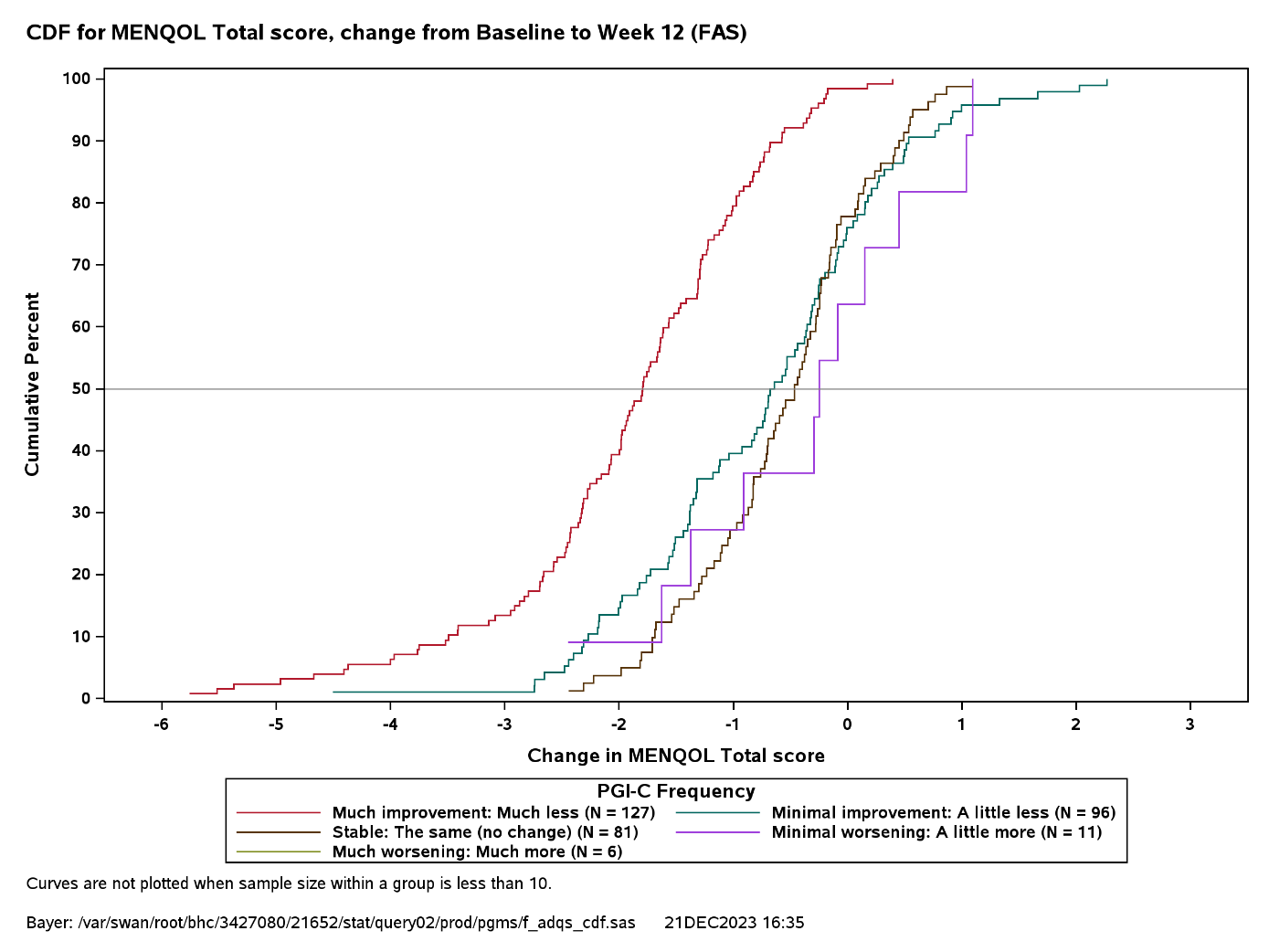


For the MENQOL Total score change from baseline to Week 12, CDF curves support the ‘minimally important’ adjusted thresholds for PGI-S Severity of -0.75 produced by logistic regression and of -1.07 for discriminant analysis (Figure S7). Both thresholds appear to correctly classify the majority of ‘improved’ participants, while correctly excluding the majority of stable participants. CDF curves support the ‘minimally important’ adjusted thresholds for PGI-C Severity of –0.84 produced by logistic regression and of –1.29 for discriminant analysis (Figure S8). Both thresholds appear to correctly classify the majority of ‘improved’ participants, while correctly excluding the majority of stable participants. However, there was overlap between curves of the ‘minimal improvement’ and ‘stable’ subjects according to the PGI-C Severity anchor.

Figure S7. CDF for MENQOL Total score, change from baseline to Week 12


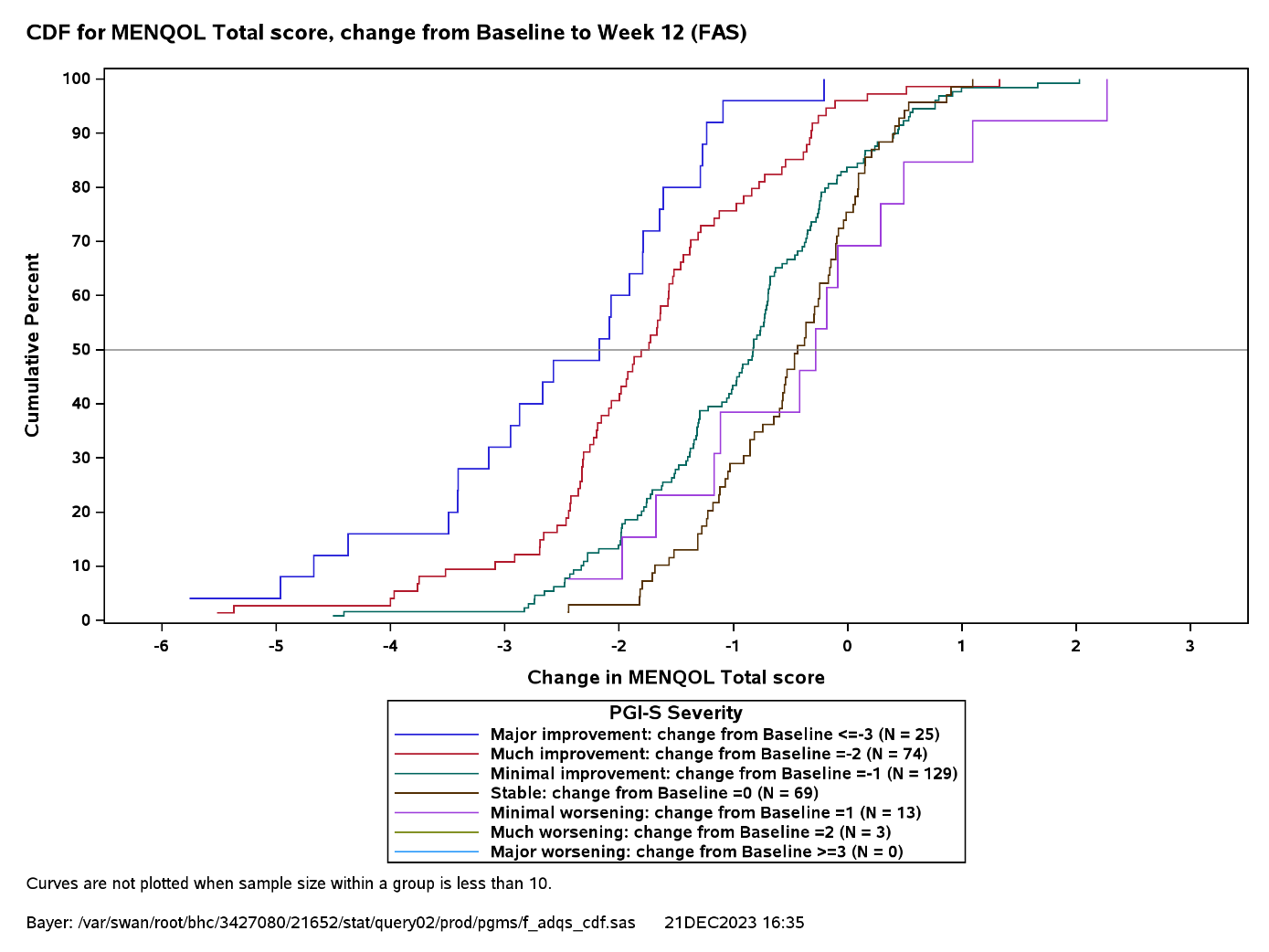


Figure S8. CDF for MENQOL Total score, change from baseline to Week 12


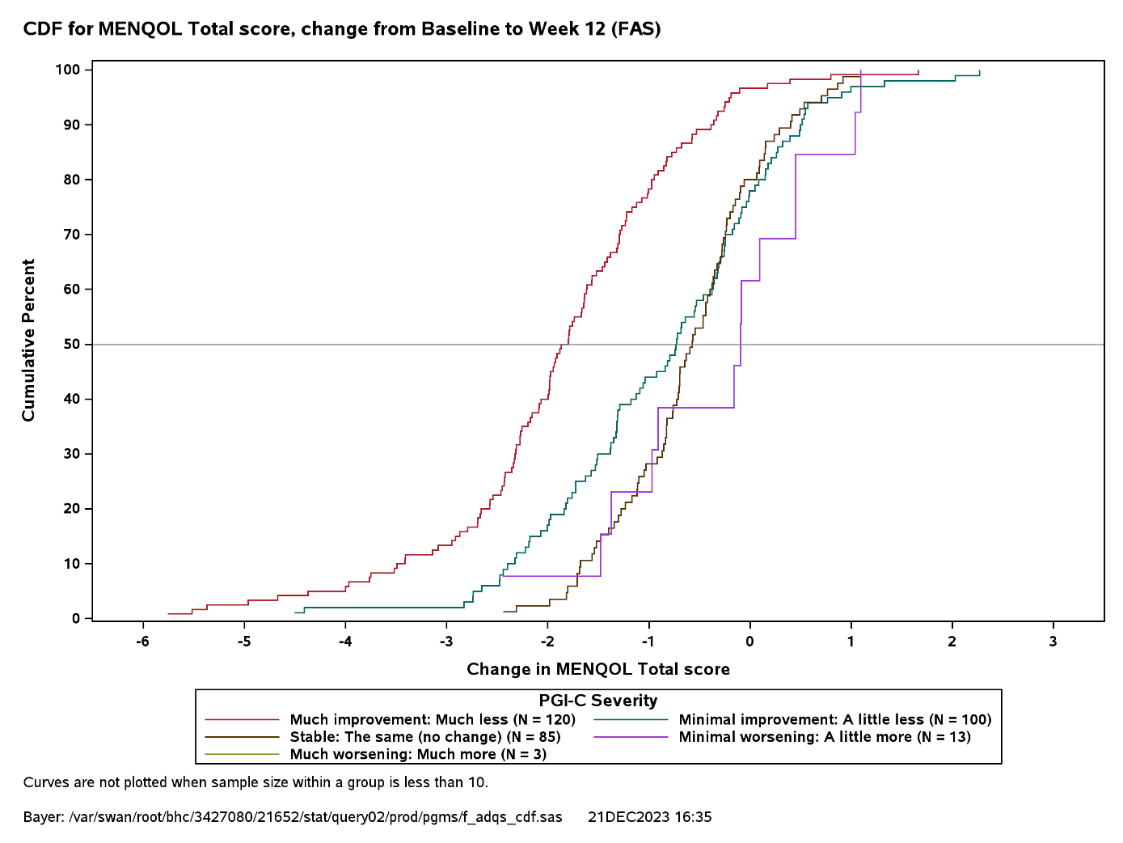

Supplement: Supplementary file 1 — Supplementary Material 1 [file 41687_2025_875_MOESM1_ESM.docx]
